# Dynamic compartment specific changes in glutathione and ascorbate levels in *Arabidopsis* plants exposed to different light intensities

**DOI:** 10.1186/1471-2229-13-104

**Published:** 2013-07-17

**Authors:** Elmien Heyneke, Nora Luschin-Ebengreuth, Iztok Krajcer, Volker Wolkinger, Maria Müller, Bernd Zechmann

**Affiliations:** 1 Department of Lothar Willmitzer, Max-Planck-Institute of Molecular Plant Physiology, Golm, 14476, Germany; 2 Institute for Electron Microscopy and Fine Structure Research, Graz University of Technology, Steyrergasse 17, Graz, 8010, Austria; 3 Institute of Plant Sciences, University of Graz, Schubertstrasse 51, Graz, 8010, Austria

**Keywords:** Arabidopsis, Ascorbate, Chloroplast, Glutathione, High light, Reactive oxygen species

## Abstract

**Background:**

Excess light conditions induce the generation of reactive oxygen species (ROS) directly in the chloroplasts but also cause an accumulation and production of ROS in peroxisomes, cytosol and vacuoles. Antioxidants such as ascorbate and glutathione occur in all cell compartments where they detoxify ROS. In this study compartment specific changes in antioxidant levels and related enzymes were monitored among Arabidopsis wildtype plants and ascorbate and glutathione deficient mutants (*vtc2-1* and *pad2-1*, respectively) exposed to different light intensities (50, 150 which was considered as control condition, 300, 700 and 1,500 μmol m^-2^ s^-1^) for 4 h and 14 d.

**Results:**

The results revealed that wildtype plants reacted to short term exposure to excess light conditions with the accumulation of ascorbate and glutathione in chloroplasts, peroxisomes and the cytosol and an increased activity of catalase in the leaves. Long term exposure led to an accumulation of ascorbate and glutathione mainly in chloroplasts. In wildtype plants an accumulation of ascorbate and hydrogen peroxide (H_2_O_2_) could be observed in vacuoles when exposed to high light conditions. The *pad2-1* mutant reacted to long term excess light exposure with an accumulation of ascorbate in peroxisomes whereas the *vtc2-1* mutant reacted with an accumulation of glutathione in the chloroplasts (relative to the wildtype) and nuclei during long term high light conditions indicating an important role of these antioxidants in these cell compartments for the protection of the mutants against high light stress.

**Conclusion:**

The results obtained in this study demonstrate that the accumulation of ascorbate and glutathione in chloroplasts, peroxisomes and the cytosol is an important reaction of plants to short term high light stress. The accumulation of ascorbate and H_2_O_2_ along the tonoplast and in vacuoles during these conditions indicates an important route for H_2_O_2_ detoxification under these conditions.

## Background

Plant metabolism strongly depends on the availability of visible light as it is the driving force for photosynthesis which converts carbon dioxide into organic compounds that are used by the plant for growth and development [1-3]. The light reaction of oxygenic photosynthesis is characterized by water oxidation and by the light driven transport of the electrons from water to NADP producing a proton gradient that facilitates the synthesis of ATP [4-7]. One key problem of this process is the constant generation of reactive oxygen species (ROS) such as singlet oxygen, superoxide hydroxyl radical and hydrogen peroxide (H_2_O_2_) [5-12]. If not detoxified these substances are capable of oxidizing membrane components and proteins and can lead to the degradation of nucleic acids, lipids, pigments, membranes, proteins, RNA, and DNA, causing mutation and eventually cell death [9-11]. The generation of ROS is naturally higher when plants are exposed to high light conditions as it overstrains the electron transport chain [7,11,13-16]. Thus, plants have to adapt to high light conditions by several metabolic and morphological changes such as reduction in the size of the light-harvesting complex [17-19], by an increase in the number of cells layers within leaves [20,21], and by the movement of chloroplasts and leaves in order to avoid the light source [19,22]. Other effective defense mechanism against high light include non-photochemical quenching which dissipates excess light as heat [22], and the detoxification of ROS by enzymes such as catalase, superoxide dismutase, oxidoreductases or antioxidants [8-10,12,22,23]. Antioxidants are molecules which are able to detoxify ROS by reducing them into less harmful substances or by inhibiting the oxidation of other molecules [24]. Besides carotenoids and α-tocopherol, which are lipohilic antioxidants within chloroplasts [10], two global antagonists against ROS are the water soluble antioxidants ascorbate and glutathione. As reducing agents they play important roles during the elimination of ROS individually or through the ascorbate glutathione cycle [10,25-28]. In opposite to carotenoids and α-tocopherol, which antioxidative effects in plants are restricted to the chloroplasts, ascorbate and glutathione and related enzymes can be found in different concentrations in the different cell compartments [29-32]. Thus ascorbate and glutathione can protect plants from oxidative damage in other cell compartments besides chloroplasts as well. Considering that H_2_O_2_ production is just as high in peroxisomes during photosynthesis as a result of glycolate oxidation in the photorespiratory pathway [15,33] and that ROS can leak from chloroplasts and peroxisomes in other cell compartments such as vacuoles and the cytosol [34,35], sufficient levels of ascorbate and glutathione in the different cell compartments are important for the plant to fight ROS during high light conditions. In Arabidopsis plants highest levels of ascorbate were found in peroxisomes and the cytosol and lowest ones in vacuoles with intermediate labeling in nuclei, mitochondria and plastids [32] whereas glutathione contents were found to be highest in mitochondria followed by nuclei, the cytosol, peroxisomes, plastids and vacuoles [31,36]. Changes in subcellular ascorbate and glutathione contents during abiotic and biotic stress are valuable stress indicators within plants and can determine the fate of the plant during situation of oxidative stress.

The aim of this study was to investigate subcellular changes in ascorbate and glutathione contents in Arabidopsis plants during the exposure to different low and high light conditions (from 50 to 1,500 μmol m^-2^ s^-1^) in order to clarify the dynamic compartment specific protection of these key antioxidants against ROS produced during high light conditions. Plants grown at 150 μmol m^-2^ s^-1^ were considered as controls (we will refer to this light intensity as control conditions throughout the text) as this light intensity is considered to be ideal for growing Arabidopsis plants and commonly used in experiments involving *Arabidopsis thaliana*. Different light intensities were applied for 4 h or 14 d in order to evaluate differences in short and long term adaptation strategies of the antioxidant system to high light intensities. Additionally, we compared dynamic changes of the antioxidants ascorbate and glutathione in ascorbate and glutathione deficient mutants, *vtc2-1* (60% less ascorbate than the wildtype) [32] and *pad2-1* (80% less glutathione than the wildtype) [36,37], respectively, in order to clarify possible compensatory effects of low ascorbate and glutathione contents by glutathione and ascorbate, respectively. Other parameters such as catalase activity and H_2_O_2_ contents, number of chloroplasts and changes in the fine structure of chloroplast were additionally monitored in order to correlate different defense and adaptation strategies of Arabidopsis plants to changes in the antioxidative protection during high and low light conditions.

## Results

**Figure 1:**
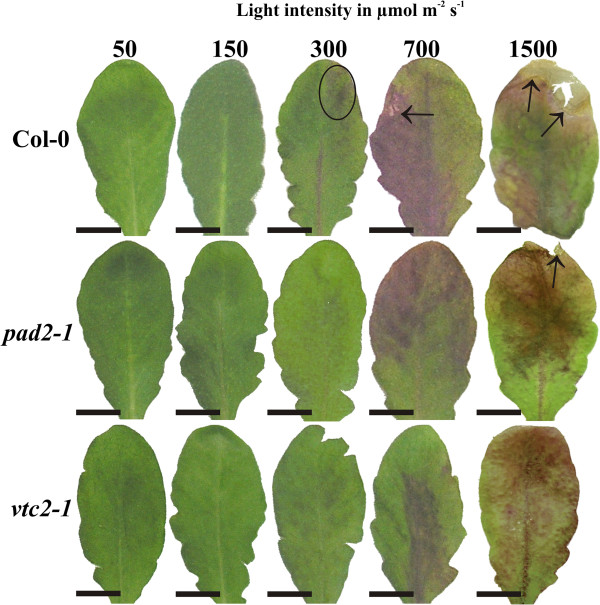
**Leaves of Col**-**0**, ***pad2****-****1 *and *vtc2****-****1 *after the exposure to different light regimes for 14 d.** Representative images of leaves from *Arabidopsis thaliana* Col-0 (first row), and the mutants *pad2-1* (second row) and *vtc2-1* (third row) grown under different light regimes for 14 d. First signs of light induced changes can be found on leaves of Col-0 grown at 300 μmol m^-2^ s^-1^ which developed dark blue stained spots on the leaf surface (circles) and on the main vascular vein. First symptoms of dark blue stained areas on the leaves of *pad2-1* and *vtc2-1* could be observed when mutants were grown under 700 μmol m^-2^ s^-1^. On leaves of the wildtype necrotic lesions (arrow) could be observed on the leaf edges at this light intensity. When leaves were exposed to 1,500 μmol m^-2^ s^-1^ large blue stained areas, advanced browning and necrotic lesions at the edge of the leaves (arrows) could be observed on Arabidopsis Col-0, the *pad2-1* and the *vtc2-1* mutant. Bar = 1 cm.

High light intensity had a major impact on the appearance and anatomy of the leaves (Figure 1; Additional file 1) and also altered the overall phenotypes of the plants. Plants exposed to high light conditions formed the same amounts of rosettes as plants grown under control conditions. Whereas fully developed leaves from older rosettes (e.g. 3rd and 4th) developed a similar size as leaves grown under control conditions leaves developed after the exposure to high light conditions remained much smaller. Thus, leaves from the 3rd and 4th rosette which were taken for the investigations described in this study had similar size and developmental stage (Figure 1). These results differ from the overall phenotypes of wildtype plants and *vtc2* mutants which were much smaller when grown for full 6 weeks under high light conditions [38] but are similar to phenotypes of the same plants exposed in adult stage for 5 days to high light conditions [39]. When leaves of *Arabidopsis thaliana* Col-0 were exposed to a light intensity of 300 μmol m^-2^ s^-1^ first blue spots -indicating the accumulation of anthocyanins in vacuoles- were visible on the leaves and on the main vascular vein (Figure 1). On leaves of the wildtype small necrotic spots on the edge of the leaves could be observed when the plants were exposed to 700 μmol m^-2^ s^-1^ (Figure 1). The *pad2-1* and *vtc2-1* mutants showed dark blue stained areas on the leaves at this light intensity but necrotic spots were not visible (Figure 1). When leaves were exposed to 1,500 μmol m^-2^ s^-1^ advanced browning and necrotic areas at the leaf edge could be observed on Arabidopsis Col-0, the *vtc2-1* and *pad2-1* mutants (Figure 1). Investigations of leaf anatomy by light microscopy revealed that wildtype plants and the *pad2-1* and *vtc2-1* mutants grown at a light intensity of 50 and 150 μmol m^-2^ s^-1^ for 14 d showed a similar ultrastructure with one tightly-packed palisade cell layer and three layers of spongy parenchyma cells with many intercellular spaces between the upper and lower epidermis cell layer (Additional file 1). At a light intensity of 300 μmol m^-2^ s^-1^ leaves of the wildtype plants and the *pad2-1* mutant contained two tightly-packed layers of palisade cells and three layers of loosely arranged spongy parenchyma cells (Additional file 1). In vacuoles of epidermis cells of leaves from wildtype plants dark stained precipitations indicating the accumulation of anthocyanins could be observed (Additional file 1). When plants were exposed to light intensities of 700 and 1,500 μmol m^-2^ s^-1^ for 14 d the accumulation of anthocyanins became more prominent and could also be found in palisade and spongy parenchyma (Additional file 1). At these light intensities a differentiation between palisade and spongy parenchyma was difficult in leaves of the wildtype and the *vtc2-1* as large intercellular spaces appeared in both cell layers. Additionally, the palisade parenchyma was reduced to one cell layer (Additional file 1). Leaves of the *pad2-1* mutants exposed to a light intensity of 700 and 1,500 μmol m^-2^ s^-1^ for 14 d showed similar anatomy as plants exposed to 300 μmol m^-2^ s^-1^ for 14 d containing two tightly-packed palisade cell layers and about 3 to 4 layers of spongy parenchyma. In general leaves of the wildtype, *pad2-1* and *vtc2-1* mutants appeared thicker at 300 and 700 μmol m^-2^ s^-1^ for 14 d in comparison to leaves exposed to the other light intensities (Additional file 1).

Subcellular changes of ascorbate and glutathione labeling were investigated by transmission electron microscopy (TEM) 4 h and 14 d after the application of different light intensities on *Arabidopsis thaliana* Col-0 plants and the *vtc2-1* and *pad2-1* mutant. The distribution of glutathione and ascorbate specific gold labeling in wildtype plants and the mutants grown at this light intensity was similar as observed in previous studies [31,32,40].

### Ascorbate

#### Exposure to different light intensities for 4 h

**Figure 2:**
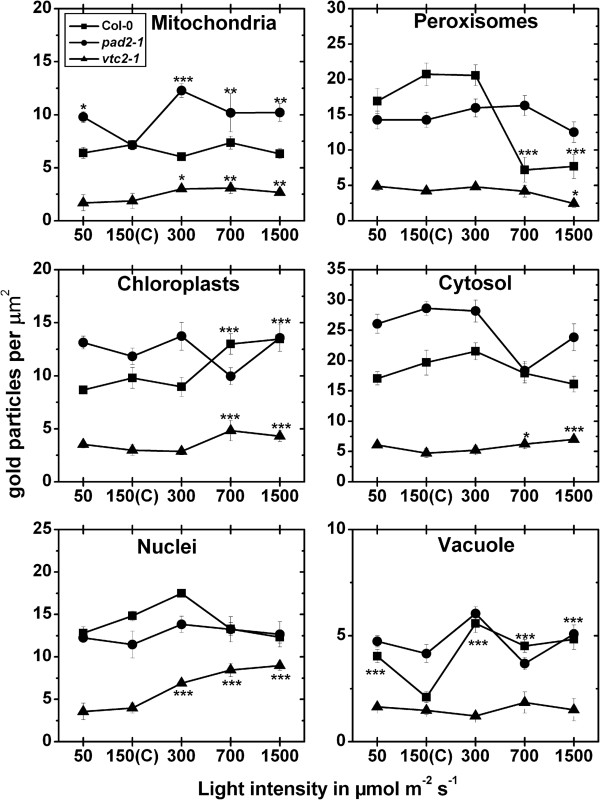
**Compartment specific ascorbate labeling density after short term light stress.** Graphs show the amounts of gold particles bound to ascorbate per μm^2^ in mesophyll cells of *Arabidopsis thaliana* Col-0 plants (black squares) and the Arabidopsis mutants *pad*2-1 (black circles) and *vtc2-1* (black triangles) after the exposure to different light intensities for 4 h. n > 20 for peroxisomes and vacuoles and n > 60 for other cell structures. Data are means with standard errors. Significant differences were calculated within one line of plants between control conditions (exposure to 150 μmol m^-2^ s^-1^) and the same line exposed to the other light intensities by using the Mann Whitney U-test; *, ** and ***, respectively, indicate significance at the 0.05, 0.01 and 0.001 levels of confidence.

**Figure 3:**
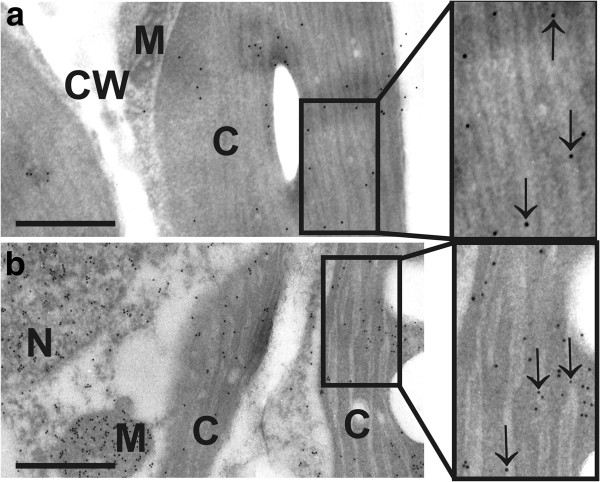
**Subcellular distribution of ascorbate and glutathione in chloroplasts of Col**-**0.** Transmission electron micrographs showing the subcellular distribution of ascorbate **(a)** and glutathione **(b)** in chloroplasts of *Arabidopsis thaliana* Col-0 plants, exposed to light intensities of 1,500 μmol m^-2^ s^-1^. Gold particles bound to ascorbate and glutathione could be found in different densities within chloroplast (C), mitochondria (M), nuclei (N), and the cytosol but not in cell walls (CW). Inside chloroplasts of control plants ascorbate and glutathione were detected in the stroma and inside the lumen of thylakoids (arrows). Bars = 0.5 μm.

In chloroplasts of wildtype plants an increase of ascobate labeling was observed when exposed to light intensities of 700 and 1,500 μmol m^-2^ s^-1^, respectively, when compared to wildtype plants grown at control conditions (Figure 2; Additional file 2). When plants were exposed to light intensities of 700 and 1500 μmol m^-2^ s^-1^ gold particles bound to ascorbate could also be observed within the lumen of thylakoids (Figure 3). In peroxisomes decreased amounts of gold particles bound to ascorbate of about 65% were detected when wildtype plants were exposed to light intensities of 700 and 1,500 μmol m^-2^ s^-1^ (Figure 2; Additional file 2). Vacuoles showed an increase in ascorbate specific labeling of up to 166% (300 μmol m^-2^ s^-1^) when wildtype plants were exposed to the different light conditions (Figure 2, Additional file 2). The *pad2-1* mutant showed changes in ascorbate contents only in mitochondria where ascorbate specific labeling was increased of up to 73% (300 μmol m^-2^ s^-1^) when mutants were exposed to the different light intensities and compared to *pad2-1* mutants grown at control conditions (Figure 2). The *vtc2-1* mutant showed significant changes in ascorbate specific labeling in all cell compartments except vacuoles when exposed to high light intensities. Mitochondria and nuclei of the *vtc2-1* mutant showed higher ascorbate contents of up to 65% and 127%, respectively, when exposed to high light intensities and compared to *vtc2-1* mutants grown in control conditions. In chloroplasts and the cytosol of the *vtc2-1* mutant increased levels of ascorbate specific labeling of up to 61% and 48% were detected when plants were exposed to 700 and 1,500 μmol m^-2^ s^-1^ light intensity (Figure 2). Peroxisomes of *vtc2-1* mutants showed significant decreased amounts of gold particles (−42%) bound to ascorbate when plants were exposed to a light intensity of 1,500 μmol m^-2^ s^-1^ (Figure 2; Additional file 2). The overall ascorbate labeling density revealed that wildtype plants showed significantly increased ascorbate contents of up to 62% in palisade cells when exposed to high light conditions (Additional file 3).

#### Exposure to different light intensities for 14 d

**Figure 4:**
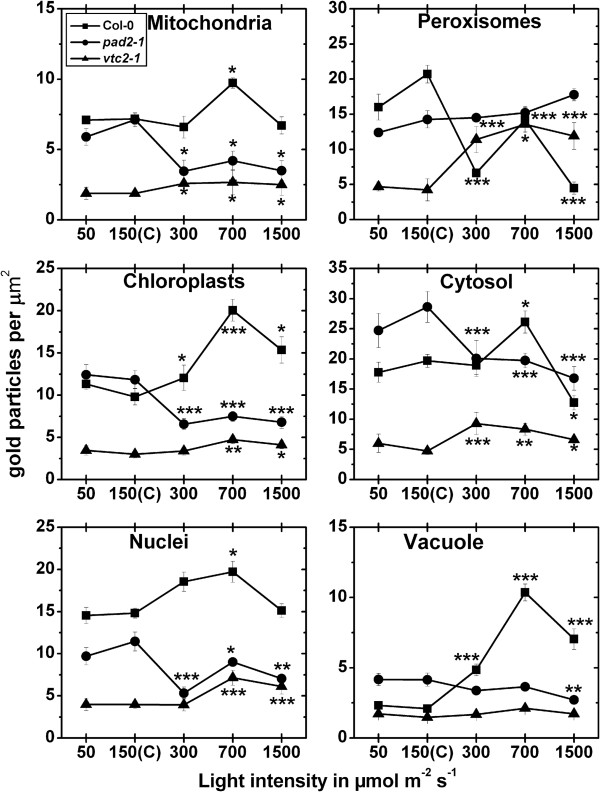
**Compartment specific ascorbate labeling density after long term light stress.** Graphs show the amounts of gold particles bound to ascorbate per μm^2^ in mesophyll cells of *Arabidopsis thaliana* Col-0 plants (black squares) and the Arabidopsis mutants *pad*2-1 (black circles) and *vtc2-1* (black triangles) after the exposure to different light intensities for 14 d. n > 20 for peroxisomes and vacuoles and n > 60 for other cell structures. Data are means with standard errors. Significant differences were calculated within one line of plants between control conditions (exposure to 150 μmol m^-2^ s^-1^) and the same line exposed to the other light intensities by using the Mann Whitney U-test; *, ** and ***, respectively, indicate significance at the 0.05, 0.01 and 0.001 levels of confidence.

Significant differences in ascorbate contents in wildtype plants were found in all cell compartments after the exposure to high light intensities (Figure 4; Additional file 4). In mitochondria a significant change (increase of 35%) could be observed when Col-0 plants were exposed to a light intensity of 700 μmol m^-2^ s^-1^ when compared to plants raised at control conditions (Figure 4). In chloroplasts, peroxisomes, and vacuoles a significant accumulation of ascorbate specific labeling of up to 104%, 78%, and 395%, respectively, could be observed when wildtype plants were exposed to high light intensities and compared to wildtype plants raised at control conditions (Figure 4; Additional file 4). In the cytosol a significant increase of ascorbate levels of 33% was observed when wildtype plants were exposed to a light intensity of 700 μmol m^-2^ s^-1^ whereas significantly decreased levels were observed when wildtype plants were exposed to 1,500 μmol m^-2^ s^-1^. In mitochondria, chloroplasts, nuclei and the cytosol ascobate specific labeling was significantly decreased between 21% and 53% when *pad2-1* mutants were exposed to light intensities of 300, 700, and 1,500 μmol m^-2^ s^-1^ and compared to *pad2-1* mutants raised at control conditions (Figure 4). In vacuoles significant changes (−35%) were only detected when plants were exposed to a light intensity of 1,500 μmol m^-2^ s^-1^ and compared to *pad2-1* mutants raised at control conditions (Figure 4; Additional file 4). In mitochondria, peroxisomes and the cytosol significant increased amounts of ascorbate of up to 42%, 221%, and 95% respectively, could be observed when *vtc2-1* mutants were exposed to high light intensities of 300, 700 and 1,500 μmol m^-2^ s^-1^, when compared to *vtc2-1* mutants raised at control conditions (Figure 4). Increased gold particle density could also be observed in chloroplasts (up to 58%) and nuclei (up to 81%) when *vtc2-1* mutants were exposed to light intensities of 700 and 1,500 μmol m^-2^ s^-1^ and were compared to the situation in *vtc2-1* mutants exposed to control light conditions (Figure 4; Additional file 4). The overall ascorbate labeling density revealed that wildtype plants and the *vtc2-1* mutants showed significantly increased ascorbate contents of up to 200% and 51%, respectively, in palisade cells when exposed to high light conditions. Decreased amounts of ascorbate of up to 38% were found in *pad2-1* exposed to high light conditions when ascorbate labeling density was calculated for a palisade cell (Additional file 3).

### Glutathione

#### Exposure to different light intensities for 4 h

**Figure 5:**
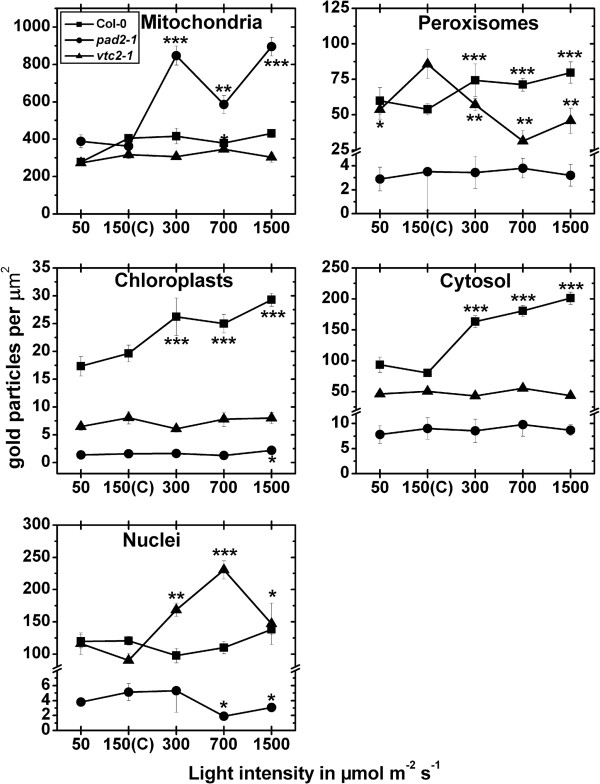
**Compartment specific glutathione labeling density after short term light stress.** Graphs show the amounts of gold particles bound to glutathione per μm^2^ in mesophyll cells of *Arabidopsis thaliana* Col-0 plants (black squares) and the Arabidopsis mutants *pad*2-1 (black circles) and *vtc2-1* (black triangles) after the exposure to different light intensities for 4 h. n > 20 for peroxisomes and n > 60 for other cell structures. No or only very few gold particles bound to glutathione were detected in vacuoles and gold particle density could therefore not be evaluated for this cell compartment. Data are means with standard errors. Significant differences were calculated within one line of plants between control conditions (exposure to 150 μmol m^-2^ s^-1^) and the same line exposed to the other light intensities by using the Mann Whitney U-test; *, ** and ***, respectively, indicate significance at the 0.05, 0.01 and 0.001 levels of confidence.

The exposure of wildtype plants to high light intensities (300, 700 and 1,500 μmol m^-2^ s^-1^) significantly increased glutathione labeling density in chloroplasts (up to 49%), peroxisomes (up to 48%), and in the cytosol (up to 151%) when compared to plants raised at control conditions (Figure 5, Additional file 5). When wildtype plants were exposed to light intensities of 700 and 1500 μmol m^-2^ s^-1^ gold particles bound to glutathione could also be observed within the lumen of thylakoids (Figure 3). The *pad2-1* mutant showed increased glutathione labeling of up to 147% in mitochondria after the exposure to high light conditions when compared to *pad2-1* mutants grown at control conditions. Whereas in chloroplasts of the *pad2-1* mutant a significant increase in gold particle density could be detected after the exposure to a light intensity of 1,500 μmol m^-2^ s^-1^ (Figure 5) decreased amounts of gold particles bound to glutathione could be observed in nuclei after the exposure to 300 and 1,500 μmol m^-2^ s^-1^ light when compared to *pad2-1* mutants grown at control conditions (Figure 5; Additional file 5). Whereas increased amounts of gold particles bound to glutathione of up to 156% could be found in nuclei of *vtc2-1* mutants after the exposure to high light intensities decreased gold labeling density was observed in peroxisomes at these light conditions (Figure 5). The overall glutathione labeling density revealed that wildtype plants and the *pad2-1* mutant showed significantly increased glutathione contents of up to 75% and 110%, respectively, in palisade cells when exposed to high light conditions (Additional file 3).

#### Exposure to different light intensities for 14 d

**Figure 6:**
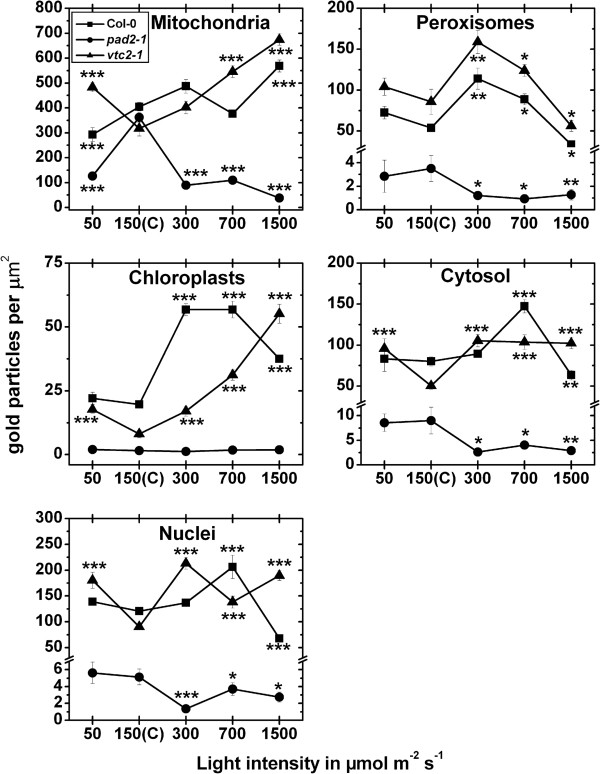
**Compartment specific glutathione labeling density after long term light stress.** Graphs show the amounts of gold particles bound to glutathione per μm^2^ in mesophyll cells of *Arabidopsis thaliana* Col-0 plants (black squares) and the Arabidopsis mutants *pad*2-1 (black circles) and *vtc2-1* (black triangles) after the exposure to different light intensities for 14 d. n > 20 for peroxisomes and n > 60 for other cell structures. No or only very few gold particles bound to glutathione were detected in vacuoles and gold particle density could therefore not be evaluated for this cell compartment. Data are means with standard errors. Significant differences were calculated within one line of plants between control conditions (exposure to 150 μmol m^-2^ s^-1^) and the same line exposed to the other light intensities by using the Mann Whitney U-test; *, ** and ***, respectively, indicate significance at the 0.05, 0.01 and 0.001 levels of confidence.

In mitochondria of the wildtype a significant decrease of 28% could be observed after the exposure to a light intensity of 50 μmol m^-2^ s^-1^, whereas a significant increase of 40% could be observed when leaves of the wildtype were exposed to 1,500 μmol m^-2^ s^-1^ for 14 d, when compared to wildtype plants exposed to control conditions (Figure 6; Additional file 6). Chloroplasts contained up to 190% more gold particles bound to glutathione when exposed to high light intensities (Figure 6). In nuclei gold particle density was strongly increased of about 71% and decreased of about 44% when leaves of the wildtype were treated with light intensities of 700 and 1,500 μmol m^-2^ s^-1^, respectively, and compared to wildtype plants grown at control conditions. Peroxisomes contained up to 112% higher glutathione specific labeling when leaves of the wildtype where exposed to 300 and 700 μmol m^-2^ s^-1^ of light (Figure 6; Additional file 6). A decrease of glutathione levels of about 38% was found in peroxisomes at a light intensity of 1,500 μmol m^-2^ s^-1^, when compared to peroxisomes in wildtype plants exposed to control conditions. The cytosol contained 84% higher gold particle labeling at 700 μmol m^-2^ s^-1^ and a significant decrease of 21% when leaves were exposed to 1,500 μmol m^-2^ s^-1^ of light (Figure 6; Additional file 6). When *pad2-1* mutants were exposed to high light conditions of 300, 700 and 1,500 μmol m^-2^ s^-1^ significantly less glutathione specific labeling was observed in mitochondria (up to 89%), nuclei (up to 74%), peroxisomes (up to 73%), and the cytosol (up to 71%) when compared to the same mutants grown at control conditions (Figure 6). In mitochondria, chloroplasts, and nuclei a significant increase in glutathione specific labeling of up to 113%, 618%, and 137%, respectively, could be observed when *vtc2-1* mutants were exposed to different light intensities and compared to *vtc2-1* mutants grown at control conditions (Figure 6; Additional file 6). Peroxisomes showed a significant increase in gold particle density of 86% and 45% and a significant decrease of 34% when *vtc2-1* mutants were exposed to light intensities of 300, 700, and 1,500 μmol m^-2^ s^-1^, respectively (Figure 6; Additional file 6). In the cytosol an increase of glutathione specific labeling between 104% and 110% was detected when plants were grown at light intensities between 300 and 1,500 μmol m^-2^ s^-1^, and compared to *vtc2-1* mutants grown at control conditions. A significant higher amount of gold particles bound to glutathione (91%) was also detected in the cytosol of the *vtc2-1* mutant when grown at a light intensity of 50 μmol m^-2^ s^-1^ and compared to the cytosol of mutants grown at control conditions (Figure 6). The overall glutathione labeling density revealed that wildtype plants and the *vtc2-1* mutant showed significantly increased glutathione contents of up to 100% and 217%, respectively, in palisade cells when exposed to high light conditions. Decreased amounts of glutathione of up to 74% were found in *pad2-1* exposed to high light conditions when glu-tathione labeling density was calculated for a palisade cell (Additional file 3).

### Chloroplast number and fine structure

**Figure 7:**
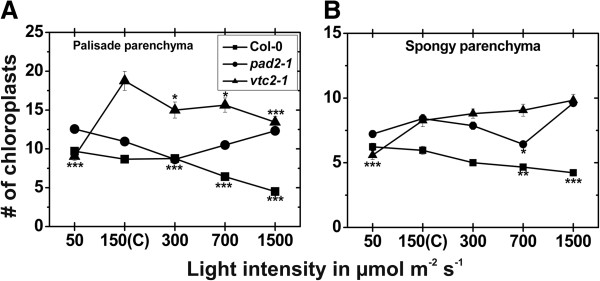
**Number of chloroplasts after the exposure to different light intensities for 14 d.** Number of chloroplasts per cell on longitudinal semithin-sections of the upper palisade cell layer **(A)** and the spongy parenchyma **(B)** in leaves of the wildtype (Col-0), the *pad2-1* and the *vtc2-1* mutants after the exposure to different light intensities for 14 d. Data are means with standard errors. Significant differences were calculated within one line of plants between control conditions (exposure to 150 μmol m^-2^ s^-1^) and the same line exposed to the other light intensities by using the Mann Whitney U-test; *, ** and ***, respectively, indicate significance at the 0.05, 0.01 and 0.001 levels of confidence. n > 100 chloroplasts per treatment.

**Figure 8:**
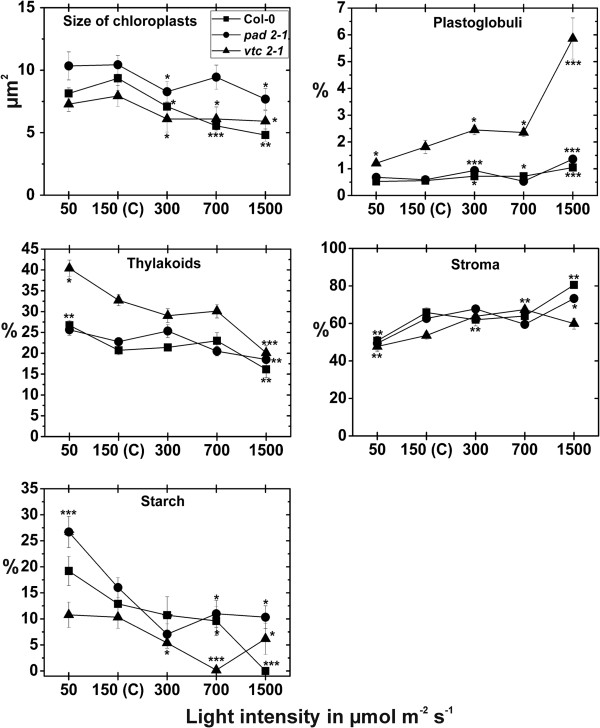
**Areas of internal chloroplast structures after the exposure to different light intensities for 14 d.** Area (in μm^2^) and percentage of areas of internal chloroplast structures detected by TEM on a longitudinal ultrathin section within the mesophyll of leaves from the wildtype (Col-0), the *pad2-1* and the *vtc2-1* mutants after the exposure to different light intensities for 14 d. Data are means with standard errors. Significant differences were calculated within one line of plants between control conditions (exposure to 150 μmol m^-2^ s^-1^) and the same line exposed to the other light intensities by using the Mann Whitney U-test; *, ** and ***, respectively, indicate significance at the 0.05, 0.01 and 0.001 levels of confidence. n > 100 chloroplasts per treatment.

When plants were raised at control conditions for 14 d the palisade cell layer of *vtc2-1* mutants contained the highest amounts of chloroplasts per cell section (18.7), followed by the *pad2-1* mutant (10.9) and the wildtype (8.7). In the spongy parenchyma *vtc2-1* and *pad2-1* mutant contained significantly more chloroplasts (8.3 and 8.4 respectively) per cell section when compared to the wildtype, which contained an average of 6 chloroplasts per cell section when plants were exposed to control conditions (Additional file 1). Chloroplast number was strongly affected by the different light treatments for 14 d. In the wildtype the amount of chloroplast was significantly decreased in the palisade cell layer (26% and 48%) and the spongy parenchyma (22% and 29%) when plants were exposed to light intensities of 700 and 1,500 μmol m^-2^ s^-1^, respectively, when compared to wildtype plants grown at control conditions (Figure 7). The *pad2-1* mutant showed significant changes in chloroplast number in the palisade cell layer and the spongy parenchyma only at a light intensity of 300 and 700 μmol m^-2^ s^-1^, respectively, where a decrease of 20% and 22% was found. The *vtc2-1* mutant showed significant decreased amounts of chloroplasts in the palisade cell layer at all light intensities when compared to *vtc2-1* mutants exposed to control conditions (Figure 7; Additional file 1). Chloroplast size was significantly lower in the wildtype, the *pad2-1* and *vtc2-1* mutant when exposed to light conditions and compared to the same plants exposed to control conditions (Figure 8). The area of thylakoid was significantly higher in the wildtype (29%) and the *vtc2-1* mutant (23%) when exposed to a light intensity of 50 μmol m^-2^ s^-1^. A decrease of thylakoid area of 22%, 19%, and 39% were observed when the wildtype, *pad2-1* and *vtc2-1* mutant, respectively, were exposed to a light intensity of 1,500 μmol m^-2^ s^-1^ and compared to the same plants exposed to control conditions (Figure 8; Additional file 7). Plastoglobuli contents were significantly increased in the wildtype (up to 88%), the *pad2-1* (up to 131%) and the *vtc2-1* (up to 223%) mutant when exposed to high light conditions. Starch contents were significantly higher (49% and 67%) in the wildtype and the *pad2-1* mutant when plants were exposed to a light intensity of 50 μmol m^-2^ s^-1^ and compared to the same plants raised at control conditions (Figure 8; Additional file 7). Significant decreased amounts of starch were found when the wildtype, the *pad*2-1 and the *vtc2-1* mutants were exposed to high light intensities. The area of the stroma was significantly lower (23% and 21%) in the wildtype and the *pad*2-1 mutant when plants were exposed to a light intensity of 50 μmol m^-2^ s^-1^ and compared to the same plants exposed to control conditions. A significant increase in stroma area of 20% and 26% was found in chloroplasts of the *vtc2-1* mutant grown at a light intensity of 300 and 700 μmol m^-2^ s^-1^, respectively. Both, the wildtype and the *pad2-1* mutant showed significant higher stroma area in the chloroplasts when exposed to a light intensity of 1,500 μmol m^-2^ s^-1^ in comparison to plants raised at control conditions (Figure 8; Additional file 7).

### Chl contents

**Figure 9:**
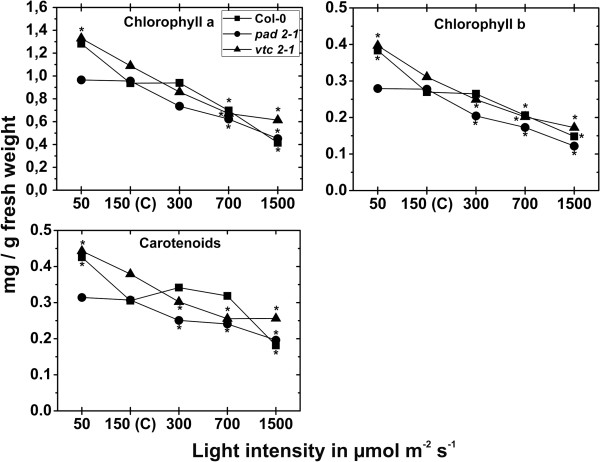
**Chl a**, **b and carotenoid contents in leaves after the exposure to different light intensities for 14 d.** Contents of Chl a, b and carotenoids in leaves of *Arabidopsis thaliana* [L.] Heynh. ecotype Columbia (Col-0), and the *pad2-1* and *vtc2-1* mutants. Data are means with standard errors. Significant differences were calculated within one line of plants between control conditions (exposure to 150 μmol m^-2^ s^-1^) and the same line exposed to the other light intensities by using the Mann Whitney U-test; *, ** and ***, respectively, indicate significance at the 0.05, 0.01 and 0.001 levels of confidence. n = 10 leaves.

Contents of Chl a, b and carotenoids of plants exposed to control conditions did not significantly differ between the wildtype (0.94, 0.27, and 0.31 mg/g fresh weight, respectively), *pad*2-1 (0.96, 0.28, and 0.31 mg/g fresh weight, respectively) and *vtc2-1* mutants (1.1, 0.31, and 0.38 mg/g fresh weight, respectively). A significant decrease in Chl a and b contents (26% and 24%, respectively) was observed when plants were exposed to 700 μmol m^-2^ s^-1^ for 14 d in comparison to wildtype plants raised at control conditions (Figure 9). When wildtype plants were exposed to 1,500 μmol m^-2^ s^-1^ chl a, b and carotenoids contents were significantly decreased of 56%, 45%, and 41%, respectively when compared to wildtype plants exposed to control conditions (Figure 9). Leaves of the *pad2-1* mutant contained significant less Chl a, b and carotenoid contents when plants were exposed to a light intensity of 300 μmol m^-2^ s^-1^ (23%, 26%, and 18%, respectively), 700 μmol m^-2^ s^-1^ (35%, 38%, and 22%, respectively), and 1,500 μmol m^-2^ s^-1^ (53%, 56%, and 36%, respectively) and compared to *pad2-1* mutants raised at control conditions (Figure 9). Chl a, b and carotenoid contents were strongly decreased in leaves of the *vtc2-1* mutants when exposed to a light intensity of 300 μmol m^-2^ s^-1^ (21%, 20%, and 20%, respectively), 700 μmol m^-2^ s^-1^ (38%, 35%, and 33%, respectively), and 1,500 μmol m^-2^ s^-1^ (44%, 45% and 32%, respectively) and compared to *vtc2-1* mutants raised at control conditions (Figure 9).

### Catalase activity and H_2_O_2_ content

**Figure 10:**
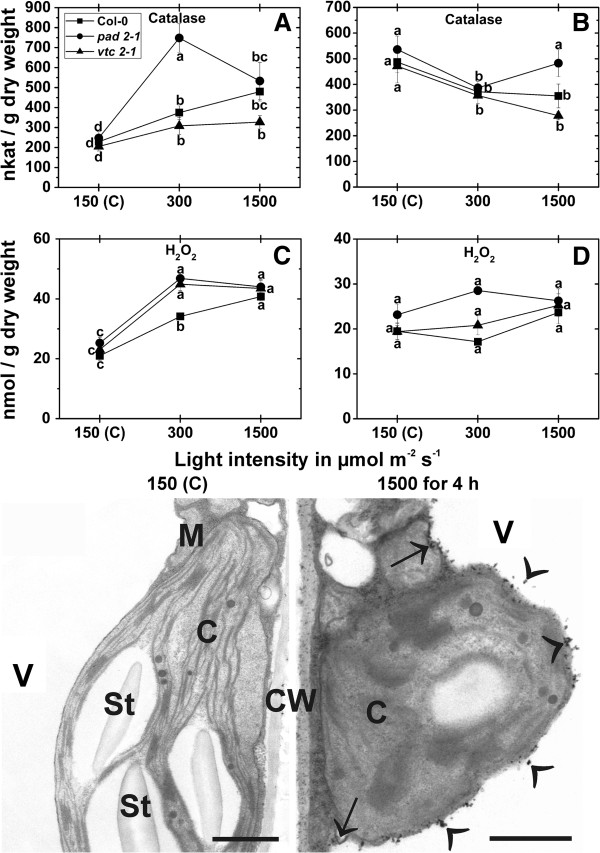
**Catalase activity**, **H**_**2**_**O**_**2 **_**contents**, **and subcellular H**_**2**_**O**_**2 **_**distribution in leaves exposed to different light regimes.** Catalase activity in nkat/g dry weight, hydrogen peroxide (H_2_O_2_) contents in nmol/g dry weight and visualized by CeCl_3_-staining on the subcellular level. Data in graphs show means with standard errors and document the activity of catalase and the amount of H_2_O_2_ detected in leaves of the wildtype (Col-0), the *pad2-1* and the *vtc2-1* mutants exposed to light conditions of 150, 300 and 1,500 μmol m^-2^ s^-1^ for 4 h **(A**, **C)** or 14 d **(B**, **D)**. Data are means with standard errors of three analysis of three pooled samples of 10 leaves from a minimum of six different plants. Different lowercase letters indicate significant differences (P < 0.05) analyzed with the Kruskal-Wallis test followed by post-hoc comparison according to Conover. TEM-micrographs show the subcellular distribution of H_2_O_2_ visualized by CeCl_3_-staining in leaves of *Arabidopsis thaliana* [L.] Heynh. ecotype Columbia (Col-0) exposed to light conditions of 150 and 1,500 μmol m^-2^ s^-1^ for 4 h. Strong CeCl_3_-staining along the tonoplast, inside vacuoles (arrowheads), the cytosol (arrows), and cell walls (CW) was observed when plants were exposed to 1,500 μmol m^-2^ s^-1^ for 4 h. Staining was only found occasionally in CW of plants raised in control conditions. C = chloroplasts with or without starch (St), M = mitochondria, V = vacuoles. Bars = 1 μm.

Catalase activity was significant increased after the exposure of wildtype plants, *pad2-1* and *vtc2-1* mutants to a light intensity of 300 μmol m^-2^ s^-1^ for 4 h (64%, 202%, and 50% respectively). A similar increase of 109% (Col-0), 122% (*pad2-1*) and 59% (*vtc2-1*) could be observed in these plants when exposed to a light intensity of 1,500 μmol m^-2^ s^-1^ for 4 h in comparison to plants exposed to control conditions (Figure 10A). Wildtype plants, *pad2-1* and *vtc2-1* mutants showed significant decreased activity of catalase (around 20%) when exposed to a light intensity of 300 μmol m^-2^ s^-1^ for 14 d and compared to the same plants raised at control conditions. A significant decrease of 27% and 41% was found in wildtype plants and the *vtc2-1* mutants, respectively when they were exposed for 14 d to a light intensity of 1,500 μmol m^-2^ s^-1^ (Figure 10B). Contents of H_2_O_2_ in plants exposed to control conditions for 4 h and 14 d did not significantly differ between the wildtype (21 and 19.5 nmol/g dry weight, respectively), *pad*2-1 (25 and 23 nmol/g dry weight fresh, respectively) and *vtc2-1* mutants (23 and 19.5 nmol/g dry weight, respectively; Figure 10 C,D). These results correlated well with the situation on the subcellular level where an accumulation of H_2_O_2_ visualized by CeCl_3_-staining within the cells could not be observed (Figure 10). After the exposure of wildtype plants, *pad2-1* and *vtc2-1* mutants to a light intensity of 300 μmol m^-2^ s^-1^ for 4 h a significant increase in H_2_O_2_ contents of 63%, 85%, and 95%, respectively, could be observed when compared to plants exposed to control conditions. A similar increase of 94% (Col-0), 74% (*pad2-1*) and 89% (*vtc2-1*) could be observed in these plants when exposed to a light intensity of 1,500 μmol m^-2^ s^-1^ for 4 h (Figure 10C). Under these conditions H_2_O_2_ accumulation visualized by CeCl_3_-staining could mainly be observed along the tonoplast and within vacuoles but also occurred in the cytosol and in cell walls in wildtype plants (Figure 10). No significant differences were observed when plants were exposed to high light conditions for 14 d (Figure 10D).

## Discussion

**Figure 11:**
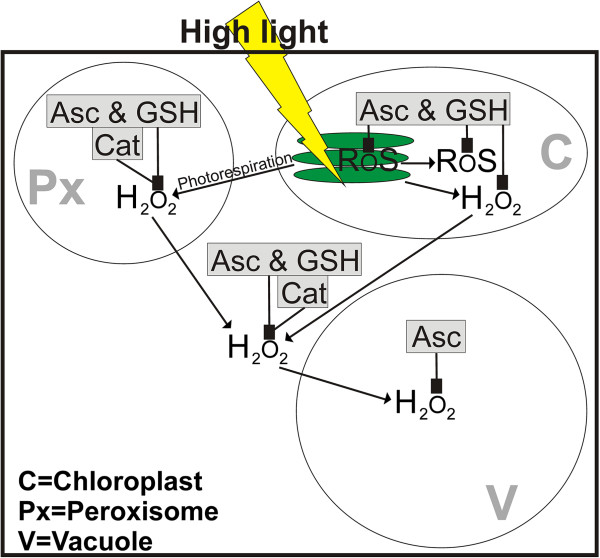
**Model of subcellular ROS accumulation and detoxification by antioxidants and catalase during high light conditions.** Line drawing proposing a model of the effects of high light stress on the subcellular accumulation of reactive oxygen species (ROS) in *Arabidopsis thaliana* with special focus on the compartment specific detoxification of hydrogen peroxide (H_2_O_2_) by ascorbate (Asc), catalase (Cat) and glutathione (GSH). High light stress (indicated by yellow thunderbold) induces the generation of ROS and H_2_O_2_ in chloroplasts (C) and in peroxisomes (Px) by overstraining the electron transport chain in thylakoids (green staples inside the chloroplast) and through photorespiration, respectively. Asc, Cat and GSH detoxify and suppress the accumulation of ROS and H_2_O_2_ in these cell compartments. With increasing light intensities H_2_O_2_ leaks from chloroplasts and peroxisomes into the cytosol and eventually into vacuoles. Whereas Asc, Cat and GSH detoxify H_2_O_2_ also in the cytosol, only Asc is involved in the detoxification of H_2_O_2_ in vacuoles where it helps to reduce phenoxyl radicals created by oxidation of phenols by H_2_O_2_[49].

Compartment specific analysis of ascorbate and glutathione in Arabidopsis plants exposed to different light conditions revealed several important aspects for the subcellular protection of these antioxidants against ROS production during high light stress. The application of a short high light stress (4 h) induced a drastic increase in overall glutathione and ascorbate labeling in wildtype plants. On the subcellular level glutathione accumulated in chloroplasts, peroxisomes and the cytosol reaching highest values in plants exposed to a light intensity of 1,500 μmol m^-2^ s^-1^, whereas ascorbate accumulated under these conditions in chloroplasts and vacuoles. These results highlight the importance of these antioxidants in chloroplasts and peroxisomes in the detoxification of ROS that are formed in these cell compartments and accumulate in the tissue within a few hours during high light conditions due to disturbance in the electron transport chain and photorespiration, respectively [23,41-48]. Nevertheless, the accumulation of H_2_O_2_ in the tissue observed in this study indicates that the ascorbate-glutathione cycle and other defense mechanisms (e.g. catalase activity which was elevated under these circumstances) were not capable of detoxifying H_2_O_2_ at a rate necessary to reduce and control H_2_O_2_ contents under high light conditions. It has been proposed recently that under such circumstances H_2_O_2_ would leak into the cytosol and vacuoles [49,50]. Such effects could be verified in this study as CeCl_3_-staining revealed the accumulation of H_2_O_2_ mainly in these two cell compartments in wildtype plants. This could be one explanation for the strong increase of ascorbate in vacuoles of wildtype plants as it is involved in the detoxification of H_2_O_2_ that diffuses into vacuoles (Figure 11) where ascorbate helps to reduce phenoxyl radicals (created by oxidation of phenols by H_2_O_2_) and is oxidized to mono- and dehydroascorbic acid which is then transported into the cytosol for reduction to ascorbic acid [49].

After the exposure of wildtype plants to high light for 14 d most cell compartments contained highest subcellular glutathione and ascorbate contents when plants were exposed to a light intensity of 300 μmol m^-2^ s^-^ or 700 μmol m^-2^ s^-1^ and a strong decrease after the exposure to a light intensity of 1,500 μmol m^-2^ s^-1^. These changes correlated with a significant reduction of Chl and carotenoid contents and the accumulation of anthocyanins after the exposure of wildtype plants to a light intensity of 700 and 1,500 μmol m^-2^ s^-1^ for 14 d. These results complement previous studies that have demonstrated increased global ascorbate levels in whole leaves exposed to long term high light conditions [38,39,51-53] and correlate well with the increase of the overall ascorbate labeling of up to 200% calculated for the whole leaf in this study. Excess light represents a potential danger to the plant as it leads to the accumulation of ROS by overstraining the reactions in chloroplasts during photosynthesis [23,43,44]. Thus, an accumulation of ascorbate and glutathione, especially in chloroplasts as observed in wildtype plants that were exposed to a light intensity of 1,500 μmol m^-2^ s^-1^ seems to be a logical consequence in order to avoid an excess production of ROS. As H_2_O_2_ contents remained at control levels in wildtype plants exposed to excess light for 14 d it seems that the observed compartment specific adaptation in ascorbate and glutathione contents as well as modification of leaf structure (increase in leaf thickness), accumulation of anthocyanins in vacuoles, decrease in Chl contents, and adaptations of chloroplast fine structure were sufficient for the adaptation of wildtype plants to excess light conditions to avoid excess production of H_2_O_2_. Chloroplasts of wildtype plants showed a strong decrease in Chl and carotenoid contents with increasing light conditions which was similar as observed under comparable conditions [38] and correlated with a decrease in thylakoid contents, chloroplast size and starch contents in the wildtype. Similar effects have been observed during drought stress in spinach and spruce tree where a decrease in starch contents, chloroplast size and an increase in plastoglobuli size could be observed [54,55]. An increase in plastoglobuli size was also observed in the present study in correlation with the exposure to high light conditions and decreasing thylakoid contents. Such a correlation seems likely during high light conditions as plastoglobuli are considered to be an important storage subcompartment of degrading thylakoid membranes [56] and play an important role in the breakdown of carotenoids [57].

In order to clarify the compartment specific importance of ascorbate and glutathione in the protection against high light conditions, subcellular ascorbate and glutathione contents were also evaluated in mutants deficient in ascorbate and glutathione contents. The glutathione deficient *pad2-1* mutant, showed a significant increase in glutathione and ascorbate contents only in mitochondria (up to 147% and 42% respectively) after the exposure to different high light regimes for 4 h which highlights the importance of glutathione and ascorbate in mitochondria for cell survival in situation of stress. The *pad2-1* mutant, which develops a phenotype similar to the wildtype, is characterized by glutathione levels similar to Col-0 in mitochondria, despite a strong drop of glutathione in all other cell compartments of up to 90% [36,37]. In previous studies we have demonstrated that distorted plant development is correlated with low glutathione contents in mitochondria as the glutathione deficient *rml1* mutant which develops a severe phenotype [58-60] shows a decrease in glutathione contents in all cell compartments including mitochondria of up to 97% [31]. Thus, we can conclude that the accumulation of antioxidants in mitochondria during high light conditions in the *pad2-1* mutant seems to be an important mechanism for the survival of plants with low glutathione contents during situation of stress. Long term exposure to high light conditions induced a strong decrease of glutathione and ascorbate contents in the *pad2-1* mutant. Only chloroplasts and peroxisomes reacted with unchanged levels of glutathione and ascorbate, respectively. Nevertheless, in comparison to the wildtype which showed a strong decrease in ascorbate contents in peroxisomes the *pad2-*1 mutant showed about 470% higher levels of ascorbate in peroxisomes after the exposure to a light intensity of 1,500 μmol m^-2^ s^-1^ for 14 d. Such effects could not be found for chloroplasts, where ascorbate contents were about 50% lower than in the wildtype which indicates that the accumulation of ascorbate in peroxisomes in the glutathione deficient *pad2-1* mutant might be an important long term strategy to detoxify H_2_O_2_ which is produced in peroxisomes under high light conditions [45-47]. This conclusion is supported by the observation that chloroplast number, fine structure, Chl contents, H_2_O_2,_ catalase behaved similar as the wildtype and the *vtc2-1* mutant to high light conditions, indicating that the *pad2-1* did not suffer more oxidative stress than the other plants. Nevertheless, the *pad2-1* mutant had thicker leaves with two palisade cell layers than the wildtype and the *vtc2-1* mutant which both had only one palisade cell layer when exposed to a light intensity of 1,500 μmol m^-2^ s^-1^ for 14 d. Thus the increase in leaf thickness at this light intensity seems to be an additional strategy of the *pad2-1* mutant in order to adapt to high light conditions. An increase in leaf thickness and higher leaf mass per unit area due to more cell layers is a commonly observed adaptation strategy to high light conditions also in other plant species [20,21].

The ascorbate deficient *vtc2-1* mutant reacted to high light conditions with a surprising strong increase of ascorbate in most cell compartments during long and short time exposure, despite alterations in ascorbate synthesis [61]. Glutathione was increased in most cell compartments after the exposure to high light conditions for 14 d but remained mostly unchanged during short term exposure. These results correlated well with the calculated overall labeling density of ascorbate and glutathione, which was significantly increased of up to 50% and 215%, respectively, when *vtc2-1* mutants were exposed to high light conditions for 14 d. These results are in line with an accumulation of total ascorbate and glutathione contents in *vtc2-2* mutants observed after the long term exposure to high light [38,39] but extend the data on the subcellular level. There it becomes obvious that the strongest accumulation of glutathione in the *vtc2-1* mutant was found in chloroplasts where an increase of 600% could be observed when plants were exposed to a light intensity of 1,500 μmol m^-2^ s^-1^ for 14 d. Additionally, ascorbate accumulated in chloroplasts under these conditions indicating that the accumulation of both ascorbate and glutathione in chloroplasts are an important adaptation strategy of the *vtc2-1* mutant to high light stress. The *vtc2-1* mutants contain about 60–80% less ascorbate under low light conditions than the wildtype [32,38] and despite an increase in ascorbate levels during high light stress, *vtc2-1* plants did not reach wildtype levels. Thus, the accumulation of glutathione in chloroplasts and also in the other cell compartments observed in this study seem to be an important adaptation strategy of the *vtc2-1* mutant especially as other mechanisms could be ruled out in this study as catalase, Chl contents, thylakoid contents, leaf anatomy, chloroplast number and the accumulation of H_2_O_2_ behaved similar to what has been observed in the wildtype and the *pad*2-1 mutant. Another interesting aspect in the *vtc2-1* mutant is the general accumulation of glutathione (and ascorbate) in nuclei at high light conditions reaching or even succeeding levels found in the wildtype. It has been proposed previously that the accumulation of reduced glutathione could serve to protect DNA and redox-sensitive nuclear proteins from oxidation, as well as driving glutaredoxin-related processes. This will influence the binding of transcription factors which will results in adaptations of gene expression patterns. Additionally, glutathione can bind to nuclear proteins and protect them from oxidation [25,62-66]. An accumulation of glutathione in nuclei, followed by a depletion from the cytosol has also been related to increased synthesis and rapid accumulation of cellular glutathione contents [65,66]. Thus, it is very well likely that the massive accumulation of glutathione in nuclei of the *vtc2-1* is additionally used to activate glutathione synthesis in order to increase cellular glutathione contents in this mutant. This hypothesis is supported by the general accumulation of glutathione in the *vtc2-1* as discussed above and by the observation that a similar accumulation has not been observed in Col-0 and *pad2-1* mutant during high light conditions.

Another interesting aspect of the adaptation of plants to high light stress was the obvious accumulation of ascorbate and glutathione inside the thylakoid lumen in wildtype, *pad2-1* and *vtc2-1* mutants exposed to high light conditions (Figure 3). Ascorbate inside the thylakoid lumen is important in respect to non-photochemical quenching which decreases the formation of ROS by dissipation of excess absorbed light as heat. One important mechanism for non-photochemical quenching is the formation of zeaxanthin to violaxanthin that is catalyzed by the enzyme violaxanthin de-epoxidase. This enzyme is located inside the thylakoid lumen and uses ascorbic acid as a reductant [67-70]. Additionally, ascorbate can be used as an alternative electron donor by photosystem II and I which is especially important in situation of stress when the linear electron transport is impaired [71,72]. Thus, the accumulation of ascorbate in the thylakoid lumen of plants exposed to high light conditions and the general increase inside the stroma highlights the importance of high ascorbate contents for the compartment specific protection of chloroplasts during high light conditions.

## Conclusions

Summing up, we can conclude that in Arabidopsis wildtype plants the accumulation of ascorbate and glutathione especially in chloroplasts, peroxisomes and the cytosol and an increased activity of catalase and enzymes involved in the ascorbate glutathione cycle are important mechanisms to protect plants against ROS produced during high light stress in the short term. Long term exposure to excess light caused an accumulation of ascorbate and glutathione in chloroplasts and several other adaptations such as the accumulation of anthocyanins in vacuoles, decrease in Chl contents and adaptations of chloroplast fine structure. Additionally, the accumulation of ascorbate in vacuoles of wildtype plants indicates an important role of this antioxidant in vacuoles for the detoxification of H_2_O_2_ leaking from peroxisomes and chloroplasts into the cytosol and vacuoles (Figure 11). The accumulation of ascorbate in peroxisomes in the glutathione deficient *pad2-1* mutant and of glutathione in the chloroplasts of the ascorbate deficient *vtc2-1* mutant relative to the wildtype Col-0 during long term exposure to high light conditions indicates an important role of these antioxidants in these cell compartments in order to protect the mutants against high light conditions.

## Methods

### Plant material

After stratification for 4 d at 4°C seeds of *Arabidopsis thaliana* [L.] Heynh. ecotype Columbia (Col-0), the glutathione and ascorbate deficient mutants *pad2-1* and *vtc2-1*, respectively, were grown on “Naturahum” potting soil (Ostendorf Gärtnereierden GmbH., Vechta, Germany) in growth chambers with 8/16 h day/night photoperiod. Day and night temperatures were set at 22°C and 18°C, respectively, the relative humidity was 60% and the plants were kept at 100% relative soil water content. Light intensity varied between 120 and 140 μmol m^-2^ s^-1^ (lower and upper leaves, respectively). Eight and six week old plants were exposed to different light intensities for 4 h and 14 d, respectively, in the same growth chamber: 1) 50 μmol m^-2^ s^-1^, 2) 150 μmol m^-2^ s^-1^, 3) 300 μmol m^-2^ s^-1^, 4) 700 μmol m^-2^ s^-1^, and 5) 1,500 μmol m^-2^ s^-1^. Different light intensities were applied by placing the plants at different distances below compact fluorescent lamps (Plug and Grow, 6400 K, white/blue spectrum; Agriculture Trading AG, Walenstadt, Switzerland). Temperature measured at the leaves was about 22°C in plants exposed to 50, 150, 300 μmol m^-2^ s^-1^, and about 24°C at plants exposed to 700 and 1,500 μmol m^-2^ s^-1^, respectively. Harvesting of the plants was performed about 4 h after the onset of the different light intensities. At the time of harvesting all plants were about 8 weeks old and all leaves were harvested from the 3th or 4th rosette. Care was taken that the leaves were about the same size, showed a similar developmental stage and that the areas chosen for further investigation were not shaded by other leaves.

### Sample preparation for TEM and immunogold labeling

#### Sample preparation

Preparation of samples for TEM, immunogold labeling of glutathione and ascorbate, and visualization of H_2_O_2_ by CeCl_3_ was performed as described previously [31,32,36,73]. Small samples of the youngest fully developed leaves close to the middle vein (about 1.5 mm^2^) from at least 3 different plants were cut on a modeling wax plate either in a drop of i) 2.5% glutaraldehyde in 0.06 M Sørensen phosphate buffer at pH 7.2 for ultrastructural investigations or ii) 2.5% paraformaldehyde, 0.5% glutaraldehyde in 0.06 M Sørensen phosphate buffer at pH 7.2 for cytohistochemical investigations or iii) 5 mM cerium chloride (CeCl_3_) in 50 mM MOPS-buffer (pH7.2) for subcellular H_2_O_2_ visualization. Samples for ultrastructural and cytohistochemical investigations were then transferred into glass vials and fixed for 90 min at room temperature (RT) in the above mentioned solutions. Samples for the visualization of subcellular H_2_O_2_ distribution were incubated with CeCl_3_ solution for 60 min and then fixed in 2.5% glutaraldehyde in 0.06 M Sørensen phosphate buffer at pH 7.2 for 90 min at RT. For ultrastructural analysis and H_2_O_2_ localization samples were then rinsed in buffer (4 times for 15 min each) and post-fixed in 1% osmium tetroxide in 0.06 M Sørensen phosphate buffer for 90 min at RT. The samples were then dehydrated in a graded series of increasing concentrations of acetone (50%, 70%, 90%, and 100%). Pure acetone was then exchanged for propylene oxide and the specimens were gradually infiltrated with increasing concentrations of Agar 100 epoxy resin (30%, 60%, and 100%) mixed with propylene oxide for a minimum of 3 h per step. Samples were finally embedded in pure, fresh Agar 100 epoxy resin (Agar Scientific Ltd, Stansted, UK) and polymerized at 60°C for 48 h. For cytohistochemical investigations samples were rinsed in 0.06 M Sørensen phosphate buffer (pH 7.2) for 4 times 15 min after fixation. They were then dehydrated in increasing concentrations of acetone (50%, 70%, and 90%) at RT for 20 min at each step. Subsequently, specimens were gradually infiltrated with increasing concentrations of LR-White resin (30%, 60% and 100%; London Resin Company Ltd., Berkshire, UK) mixed with acetone (90%) for a minimum of 3 h per step. Samples were finally embedded in pure, fresh LR-White resin and polymerized at 50°C for 48 h in small plastic containers under anaerobic conditions. Ultrathin sections (80 nm) were cut with a Reichert Ultracut S ultramicrotome (Leica Microsystems, Vienna, Austria).

#### Determination of chloroplast number and their fine structures

Changes in the number of chloroplasts and their inner structures were evaluated according to Zechmann et al. [74] by investigating four different leaf samples from wildtype plants and mutants grown in different light intensities for 14 d. Chloroplast number and inner structures were not further evaluated in plants exposed to different light intensities for 4 h as no obvious differences could be found. An Olympus AX70 light microscope (Olympus, Life and Material Science Europa GmbH, Hamburg, Germany) with a 40× objective lens (n.a. 0.5-1.35) was used to determine the number of sectioned chloroplasts in the palisade cell layer and the spongy parenchyma by counting the chloroplasts per cell on 4 semithin cross-sections (3 μm) for each replicate sample. A minimum of 100 cells per leaf-type were examined to calculate the number of sectioned chloroplasts in the cells. Ultrathin sections were investigated with the TEM to determine changes in the ultrastructure of the chloroplasts including the thylakoid-system, starch grains, and plastoglobuli. These structures were then analyzed as digital images using the program Optimas 6.5.1 (BioScan Corp.). A minimum of 20 sectioned chloroplasts from at least 10 different cells from four different samples per leaf-type were examined.

#### Immunogold labeling of glutathione and ascorbate

Immunogold labeling of glutathione and ascorbate was done according to Zechmann et al. [32,36] with ultrathin sections on coated nickel grids with the automated immunogold labeling system Leica EM IGL (Leica, Microsystems, Vienna, Austria). The ideal dilutions and incubation times of the primary (anti-ascorbate IgG; Abcam plc, Cambridge, UK; anti-glutathione rabbit polyclonal IgG, Millipore Corp., Billerica, MA, USA) and secondary antibodies (goat anti rat IgG and goat anti rabbit both from British BioCell International, Cardiff, UK) were determined in preliminary studies by evaluating the labeling density after a series of labeling experiments. The final dilution of primary and secondary antibodies used in this study showed a minimum of background labeling outside the sample with a maximum of specific labeling in the sample. The sections were blocked for 20 min with 2% bovine serum albumine (BSA, Sigma-Aldrich, St. Louis, MO, USA) in phosphate buffered saline (PBS, pH 7.2) and then treated with the primary antibodies against ascorbate diluted 1:300 in PBS containing 1% BSA and glutathione diluted 1:50 in PBS containing 1% goat serum for 2 h at RT. After a short rinse in PBS (3 times 5 min each), samples were incubated with a 10 nm gold-conjugated secondary antibodies (goat and rat IgG for ascorbate labeling and goat anti rabbit IgG for glutathione labeling) diluted 1:50 (for sections incubated with the glutathione antibody) and 1:100 (for sections incubated with the ascorbate antibody) in PBS for 90 min at RT. After a short wash in PBS (3 times 5 min), and distilled water (2 times 5 min) labeled grids were either immediately observed in a Philips CM10 transmission electron microscope or post stained with uranyl-acetate (2% dissolved in aqua bidest) for 15 s.

Micrographs of randomly photographed immunogold labeled sections in palisade parenchyma cells were digitized and gold particles were counted automatically using the software package Cell D with the particle analysis tool (Olympus, Life and Material Science Europa GmbH, Hamburg, Germany) in different visually identified and manually traced cell structures (mitochondria, plastids, nuclei, peroxisomes, the cytosol, vacuoles). Unspecific background labeling was determined on the sections (outside the specimen) and subtracted from the values obtained in the sample. A minimum of 20 (peroxisomes and vacuoles) to 60 (other cell structures) sectioned cell structures of at least 15 different cells were analyzed for gold particle density per sample. The obtained data were statistically evaluated using Statistica (Stat-Soft Europe, Hamburg, Germany).

Several negative controls were made to support the specificity of the immunogold procedure. Negative controls were treated either with (i) gold conjugated secondary antibody (goat anti rat IgG for ascorbate and goat anti rabbit IgG for glutathione) without prior incubation of the section with the primary antibodies, (ii) non specific secondary antibody (goat anti rabbit IgG for ascorbate and goat anti rat IgG for glutathione), (iii) preimmune serum instead of the primary antibody and (iv) primary antibody against ascorbate and glutathione pre-adsorbed with an excess of reduced and oxidized ascorbate and glutathione, respectively for 2 h prior to labeling of the sections. For the latter a solution containing either 10 mM of ascorbic acid, dehydroascorbic acid, reduced or oxidized glutathione was incubated with or without 0.5% glutaraldehyde for 1 h. When glutaraldehyde was used then its excess was saturated by incubation for 30 min in a solution of 1% (w/v) BSA. The resulting solutions were both used in independent experiments to saturate the anti-ascorbate and glutathione antibodies for 2 h prior to its use in the immunogold labeling procedure described above. Labeling on sections treated as negative controls showed no or only very little gold particles bound to ascorbate and glutathione which was similar to previous results obtained by using the same methods in different plant species [31,32]. The specificity and accuracy of the immunogold localization method for ascorbate and glutathione used in this study has been demonstrated in detail in previous works [31,32,36]. The immunogold localization of ascorbate in mutants deficient in ascorbate (*vtc2-1* and *vtc2-2*) revealed a strong decrease of subcelluar ascorbate specific labeling between 50 to 60% when compared to *Arabidopsis thaliana* Col-0 plants. This data correlated well with biochemical measurements which revealed a similar decrease of ascorbate contents in whole leaves of these mutants [32]. The specificity and accuracy of the immunogold labeling method for glutathione was demonstrated on glutathione deficient mutants *pad2-1* and *rml1* which both showed a strong decrease of compartment specific glutathione labeling of up to 91% and 98%, respectively. This data correlated well with biochemical measurements of glutathione in these mutants revealing a similar decrease in whole leaves of these mutants [37,59,60]. Further studies using rapid fixation methods (e.g. high pressure freezing, microwave assisted fixation) revealed that ascorbate and glutathione were not redistributed or washed out during chemical fixation at RT which takes 90 min as labeling density and the ratio of labeling between cell compartments remained the same when these methods were used [31,32].

### Biochemical investigations

#### Determination of Chl a/b and carotenoids

Plant tissues were frozen and ground in liquid nitrogen. Chl and carotenoids were extracted with 100% acetone in darkness at 4°C for 20 min. The homogenate was centrifuged and pigment content was quantified spectrophotometrically by measuring the absorbance at 663, 645 and 470 nm on a UV-spectrophotometer (Hitachi U-3000). Pigment content was calculated as described previously [75].

#### Activity of catalase

Catalase activity was measured as previously described [76]. Leaf material was frozen in liquid nitrogen, freeze dried and ground with an oscillating mill (Retsch MM400). 75 mg insoluble polyvinylpyrrolidone (PVP) was added to 20 mg plant material and the powder was extracted into 2.2 ml 0.1 M NaH_2_PO_4_ (pH 7.5), 1 mM EDTA. Samples were centrifuged for 10 min at 14000 rpm at 4°C and the supernatant was used for further investigations. Catalase activity was measured with a photometer (Hitachi U3000) as the absorbance decrease at 240 nm (ϵ_240_ = 0.04 mM^-1^ cm^-1^) at 25°C in 50 mM KH_2_ PO_4_ and 40 mM H_2_O_2_ against reagent blank.

#### Content of H_2_O_2_

H_2_O_2_ contents were measured as previously described [77]. Leaf material was frozen in liquid nitrogen, freeze dried and ground with an oscillating mill (Retsch MM400). 50 mg plant material was extracted in 1 ml 0.2 M perchloric acid and centrifuged at 14000 rpm for 10 min at 4°C. The supernatant was neutralized with a 4 M sodium hydroxide solution (pH 7.5) and centrifuged at 14000 rpm for 10 min at 4°C. The supernatant was used for further analysis as described previously [78]. Briefly, 300 μl of the supernatant was mixed with 100 μl of a known amount of hydrogen peroxide, 100 μl 2% potassium iodide solution, 100 μl of 2 M hydrochloric acid. The mixture was incubated for 10 min on a shaker and then 50 μl of 0.01% toluidine blue indicator solution followed by 200 μl 2 M sodium acetate solution and 150 μl distilled water were added. Absorbance was measured with a photometer (Hitachi U3000) at 628 nm against a reagent blank.

## Abbreviations

BSA: Bovine serum albumin; H2O2: Hydrogen peroxide; pad: Phytoalexin deficient; PBS: Phosphate buffered saline; rml: Root meristemless; ROS: Reactive oxygen species; RT: Room temperature; TEM: Transmission electron microscopy; vtc: Vitamin C1

## Competing interests

The authors declare that they have no competing interests.

## Authors’ contributions

BZ conceived of the study and participated in its design and coordination, participated in the electron and light microscopical work and drafted the manuscript together with the co-authors. EH, NL and IK performed electron and light microscopical studies, and performed quantitative and statistical analysis of the data. NL and VW performed biochemical studies and statistical analysis of the data. MM coordinated and participated in biochemical studies and performed statistical analysis of the data. All authors read and approved the final manuscript.

## Supplementary Material

Additional file 1 **Leaf sections of Col-0**, ***pad2****-****1 *and *vtc2****-****1 *after the exposure to different light regimes for 14 d.** Representative light microscopical images of leaf sections from Arabidopsis thaliana Col-0 (first row), and the mutants *pad2-1* (second row) and *vtc2-1* (third row) grown under different light regimes for 14 d. Similar structure was found in leaves of plants grown at a light intensity of 50 and 150 μmol m^-2^ s^-1^ which developed one palisade cell layer and 3 layers of spongy parenchyma cells between the upper and lower epidermis. Plants exposed to a light intensity of 300 μmol m^-2^ s^-1^ developed two palisade cell layers. This leaf structure remained the same within leaves of the *pad2-1* mutant exposed to a light intensity of 700 and 1,500 μmol m^-2^ s^-1^. Leaves of the wildtype and the *vtc2-1* mutant exposed to a light intensity of 700 and 1,500 μmol m^-2^ s^-1^ was characterized by an increase in number and size of intercellular spaces thus rendering the differentiation between palisade and spongy parenchyma difficult. Dark stained vacuoles (arrows) indicate the accumulation of anthocyanins which can be best seen in wildtype plants exposed to a light intensity of 700 and 1,500 μmol m^-2^ s^-1^ and in mutants exposed to a light intensity of 1,500 μmol m^-2^ s^-1^. Bars = 50 μm. Click here for file

Additional file 2: **TEM**-**micrographs of ascorbate labeling in plants grown under different light regimes for 4 h.** Representative transmission electron micrographs showing gold particles bound to ascorbate on leaf sections from Arabidopsis thaliana Col-0 (first row), and the mutants *pad2-1* (second row) and *vtc2-1* (third row) grown under different light regimes for 4 h. Bars = 1 μm. C = chloroplasts with or without starch (St), IS = intercellular spaces, M = mitochondria, N = nuclei, Px = peroxisomes, V = vacuoles. Click here for file

Additional file 3 **Graph showing the overall ascorbate and glutathione labeling in plants grown under different light regimes.** Ascorbate and glutathione content per cell was obtained according to Koffler et al. [79] using the corresponding labeling density in Col-0, *pad2-1* and *vtc2-1* exposed to different light regimes for 4 h (short day) and 14 d (long day) and the relative compartment volume from the leaf center of older leaves [79], where the sum runs over all compartments. Values represent the amounts of gold particles per μm^2^ within a palisade cell. Significant differences were calculated within one line of plants between control conditions (exposure to 150 μmol m^-2^ s^-1^) and the same line exposed to the other light intensities by using the Mann Whitney U-test; *, ** and ***, respectively, indicate significance at the 0.05, 0.01 and 0.001 levels of confidence. Click here for file

Additional file 4 **TEM**-**micrographs of ascorbate labeling in plants grown under different light regimes for 14 d.** Representative transmission electron micrographs showing gold particles bound to ascorbate on leaf sections from Arabidopsis thaliana Col-0 (first row), and the mutants *pad2-1* (second row) and *vtc2-1* (third row) grown under different light regimes for 14 d. Bars = 1 μm. C = chloroplasts with or without starch (St), IS = intercellular spaces, M = mitochondria, N = nuclei, Px = peroxisomes, V = vacuoles. Click here for file

Additional file 5 **TEM**-**micrographs of glutathione labeling in plants grown under different light regimes for 4 h.** Representative transmission electron micrographs showing gold particles bound to glutathione on leaf sections from Arabidopsis thaliana Col-0 (first row), and the mutants *pad2-1* (second row) and *vtc2-1* (third row) grown under different light regimes for 4 h. Bars = 1 μm. C = chloroplasts with or without starch (St), M = mitochondria, N = nuclei, Px = peroxisomes, V = vacuoles. Click here for file

Additional file 6 **TEM**-**micrographs of glutathione labeling in plants grown under different light regimes for 14 d.** Representative transmission electron micrographs showing gold particles bound to glutathione on leaf sections from Arabidopsis thaliana Col-0 (first row), and the mutants *pad2-1* (second row) and *vtc2-1* (third row) grown under different light regimes for 14 d. Bars = 1 μm. C = chloroplasts with or without starch (St), M = mitochondria, N = nuclei, Px = peroxisomes, V = vacuoles. Click here for file

Additional file 7 **TEM**-**micrographs of chloroplasts from plants grown under different light regimes for 14 d.** Representative transmission electron micrographs of chloroplasts from Arabidopsis thaliana Col-0 (first row), and the mutants *pad2-1* (second row) and *vtc2-1* (third row) grown under different light regimes for 14 d. Bars = 1 μm. C = chloroplasts with or without starch (St) and plastoglobuli (arrowheads), IS = intercellular spaces, M = mitochondria, N = nuclei, Px = peroxisomes, V = vacuoles. Click here for file
