# Supplementary figures and images for: Dynamic compartment specific changes in glutathione and ascorbate levels in Arabidopsis plants exposed to different light intensities
Source: BMC Plant Biol. 2013 Jul 17;13:104. doi: 10.1186/1471-2229-13-104 (PMC3728233; doi:10.1186/1471-2229-13-104)

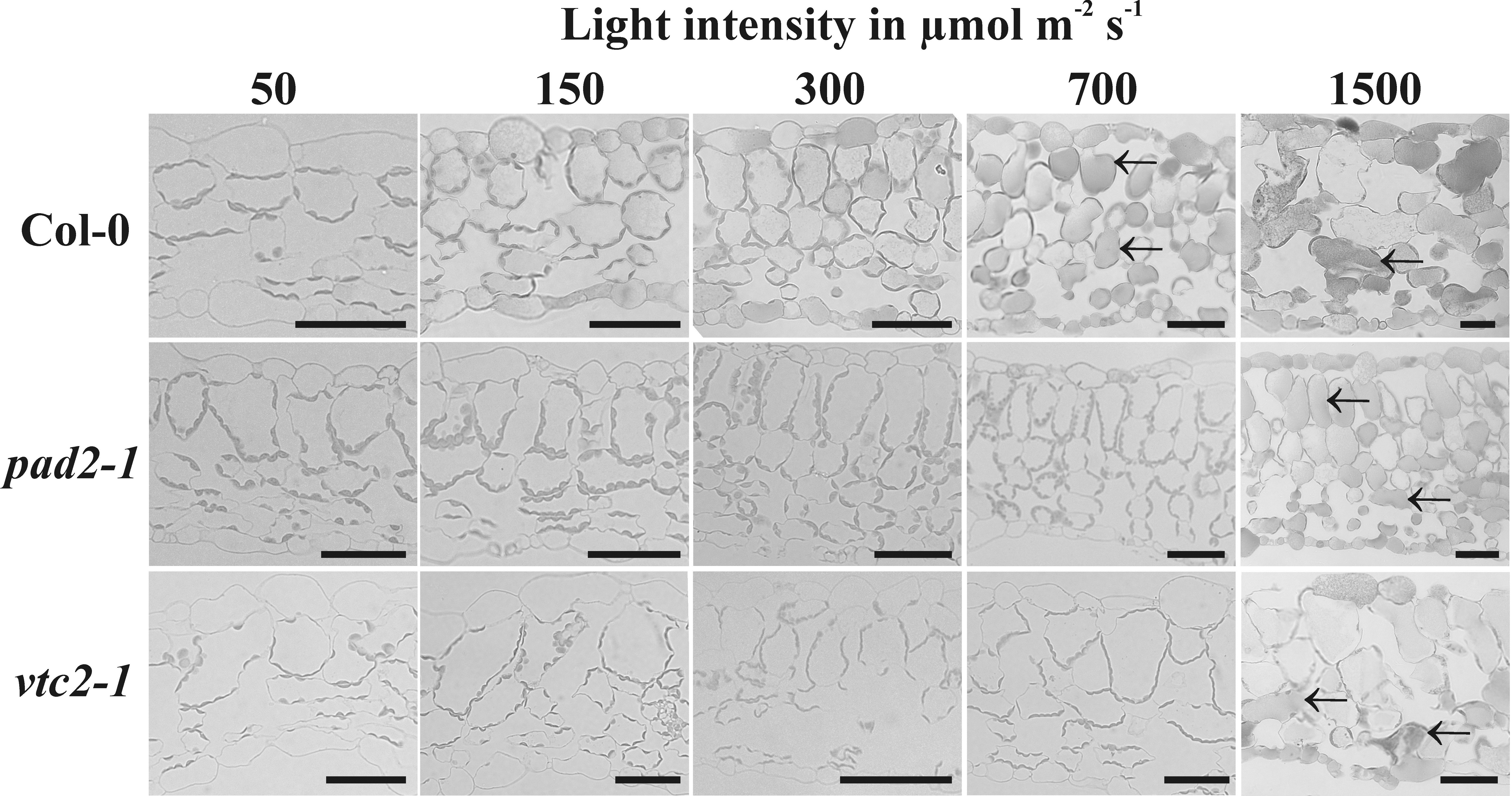

Supplement: Additional file 1 — Leaf sections of Col-0, pad2-1 and vtc2-1 after the exposure to different light regimes for 14 d. Representative light microscopical images of leaf sections from Arabidopsis thaliana Col-0 (first row), and the mutants pad2-1 (second row) and vtc2-1 (third row) grown under different light regimes for 14 d. Similar structure was found in leaves of plants grown at a light intensity of 50 and 150 μmol m-2 s-1 which developed one palisade cell layer and 3 layers of spongy parenchyma cells between the upper and lower epidermis. Plants exposed to a light intensity of 300 μmol m-2 s-1 developed two palisade cell layers. This leaf structure remained the same within leaves of the pad2-1 mutant exposed to a light intensity of 700 and 1,500 μmol m-2 s-1. Leaves of the wildtype and the vtc2-1 mutant exposed to a light intensity of 700 and 1,500 μmol m-2 s-1 was characterized by an increase in number and size of intercellular spaces thus rendering the differentiation between palisade and spongy parenchyma difficult. Dark stained vacuoles (arrows) indicate the accumulation of anthocyanins which can be best seen in wildtype plants exposed to a light intensity of 700 and 1,500 μmol m-2 s-1 and in mutants exposed to a light intensity of 1,500 μmol m-2 s-1. Bars = 50 μm. [file 1471-2229-13-104-S1.jpeg]

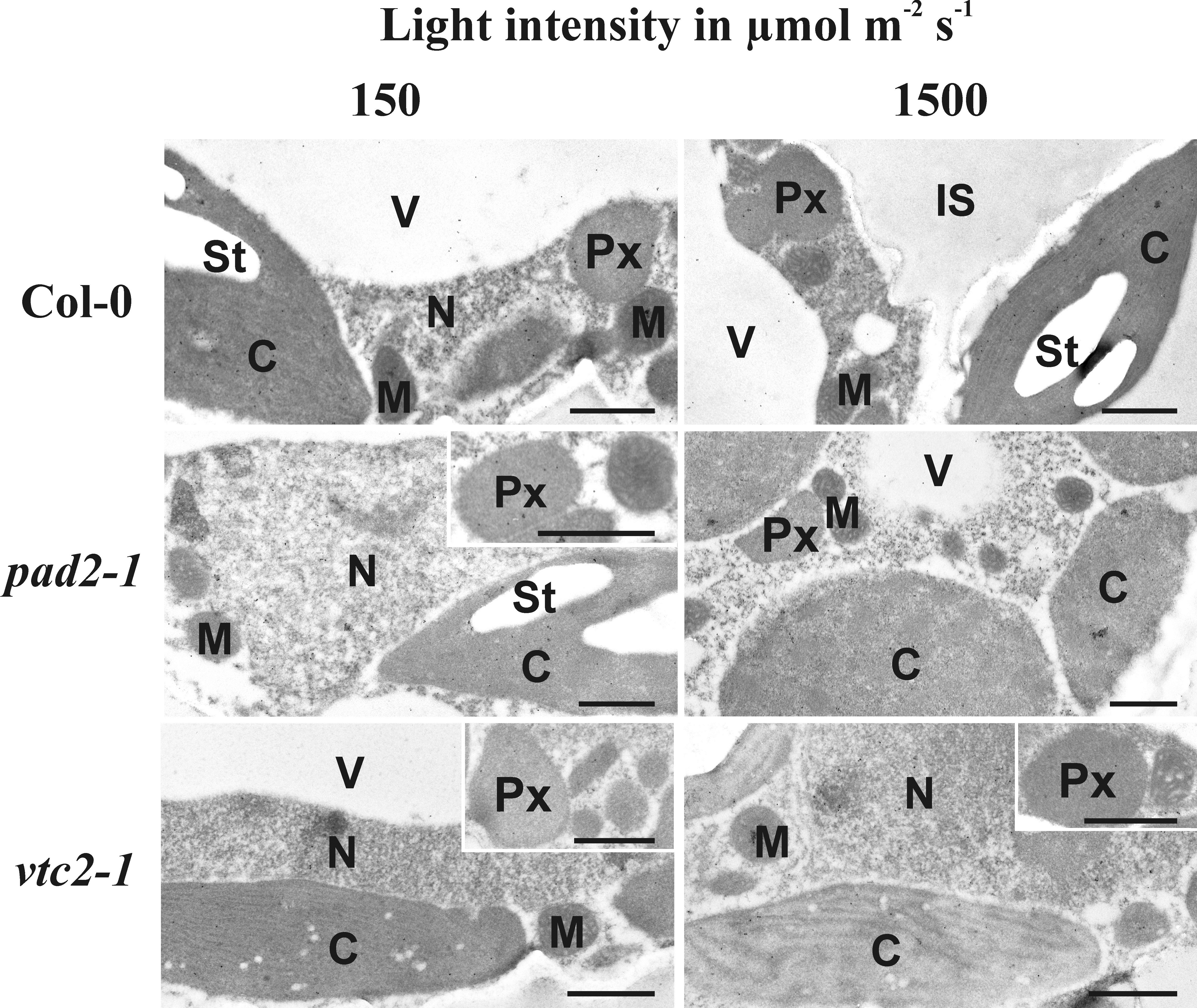

Supplement: Additional file 2: — TEM-micrographs of ascorbate labeling in plants grown under different light regimes for 4 h. Representative transmission electron micrographs showing gold particles bound to ascorbate on leaf sections from Arabidopsis thaliana Col-0 (first row), and the mutants pad2-1 (second row) and vtc2-1 (third row) grown under different light regimes for 4 h. Bars = 1 μm. C = chloroplasts with or without starch (St), IS = intercellular spaces, M = mitochondria, N = nuclei, Px = peroxisomes, V = vacuoles. [file 1471-2229-13-104-S2.jpeg]

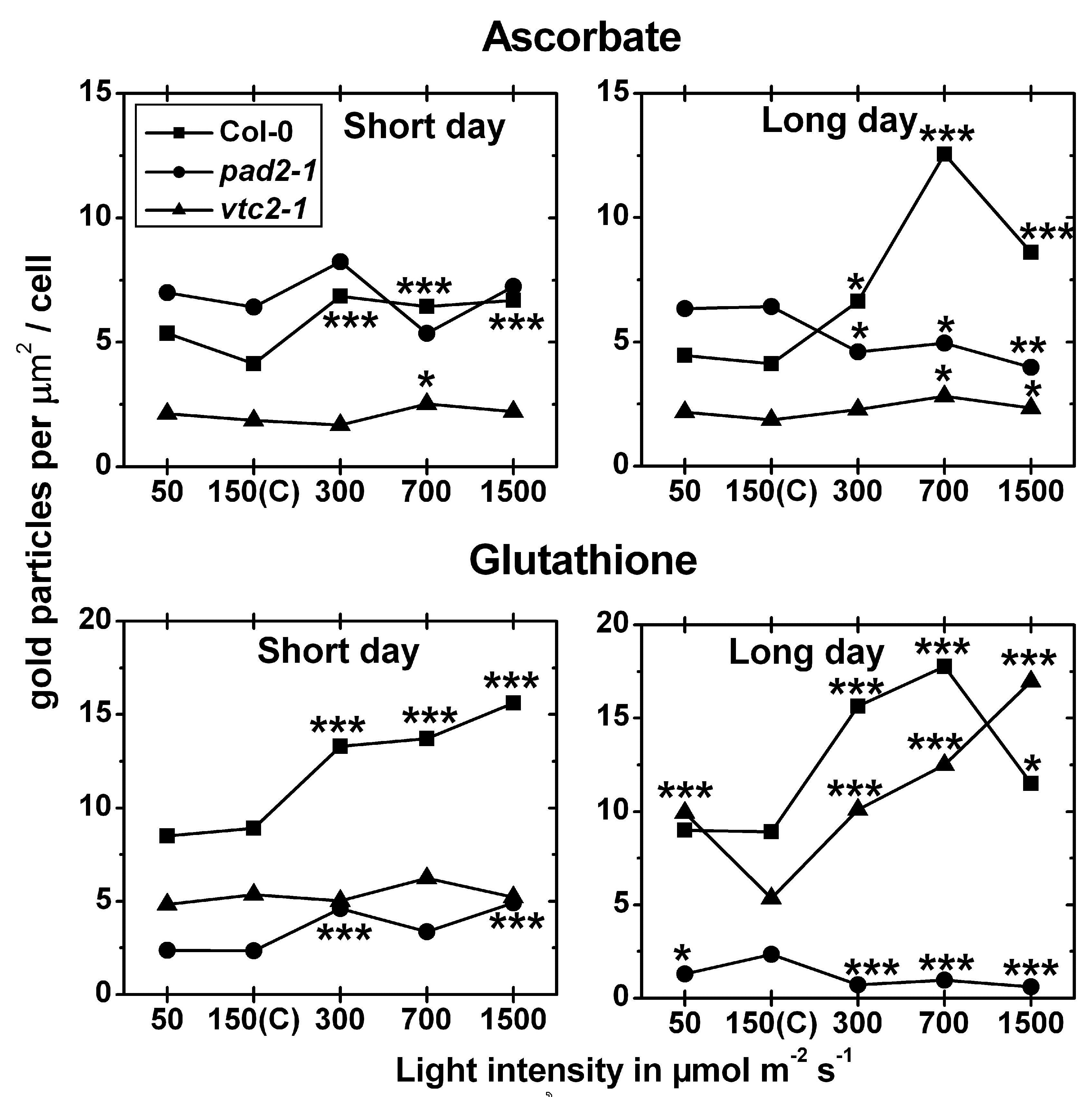

Supplement: Additional file 3 — Graph showing the overall ascorbate and glutathione labeling in plants grown under different light regimes. Ascorbate and glutathione content per cell was obtained according to Koffler et al. [79] using the corresponding labeling density in Col-0, pad2-1 and vtc2-1 exposed to different light regimes for 4 h (short day) and 14 d (long day) and the relative compartment volume from the leaf center of older leaves [79], where the sum runs over all compartments. Values represent the amounts of gold particles per μm2 within a palisade cell. Significant differences were calculated within one line of plants between control conditions (exposure to 150 μmol m-2 s-1) and the same line exposed to the other light intensities by using the Mann Whitney U-test; *, ** and ***, respectively, indicate significance at the 0.05, 0.01 and 0.001 levels of confidence. [file 1471-2229-13-104-S3.jpeg]

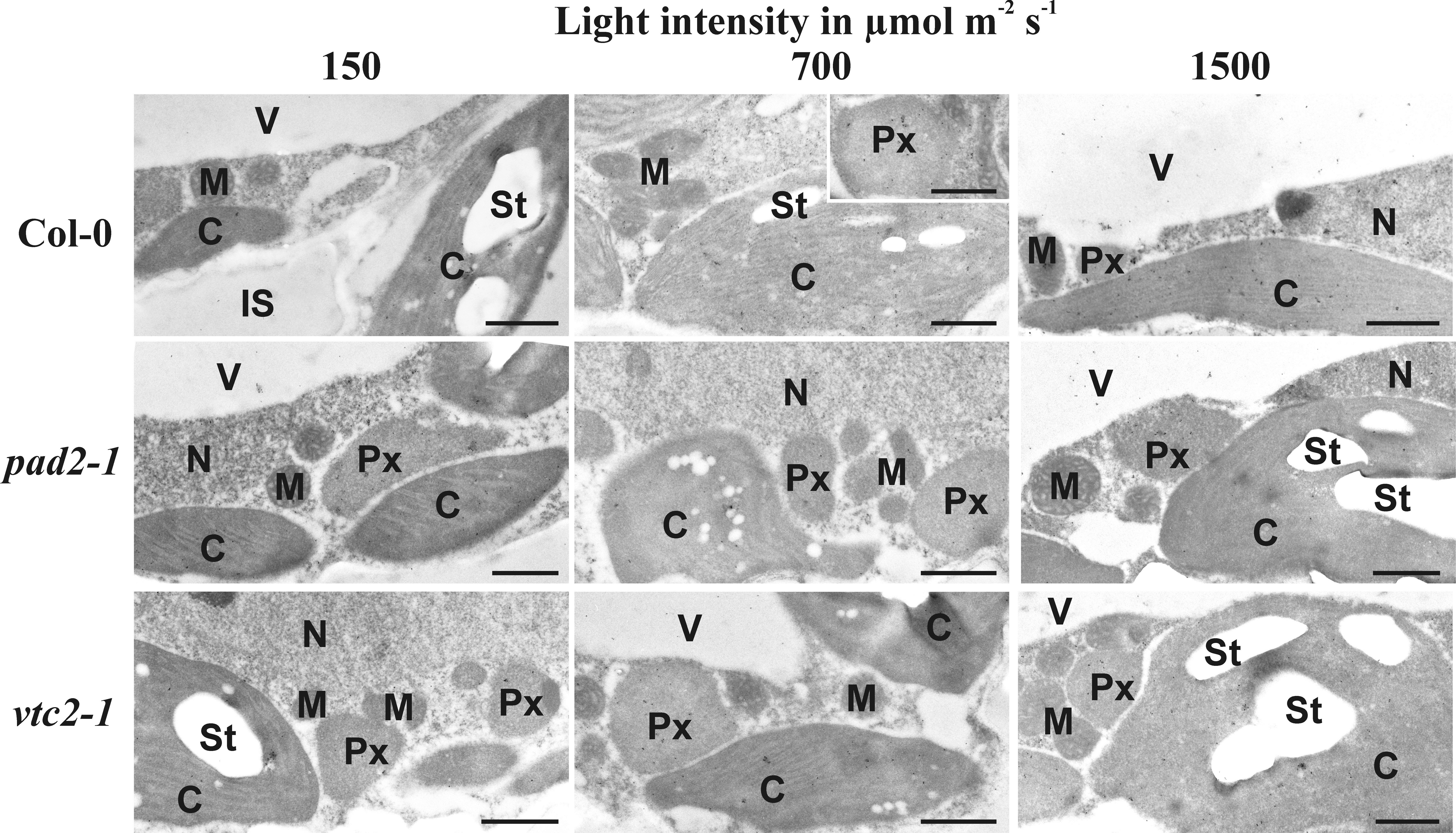

Supplement: Additional file 4 — TEM-micrographs of ascorbate labeling in plants grown under different light regimes for 14 d. Representative transmission electron micrographs showing gold particles bound to ascorbate on leaf sections from Arabidopsis thaliana Col-0 (first row), and the mutants pad2-1 (second row) and vtc2-1 (third row) grown under different light regimes for 14 d. Bars = 1 μm. C = chloroplasts with or without starch (St), IS = intercellular spaces, M = mitochondria, N = nuclei, Px = peroxisomes, V = vacuoles. [file 1471-2229-13-104-S4.jpeg]

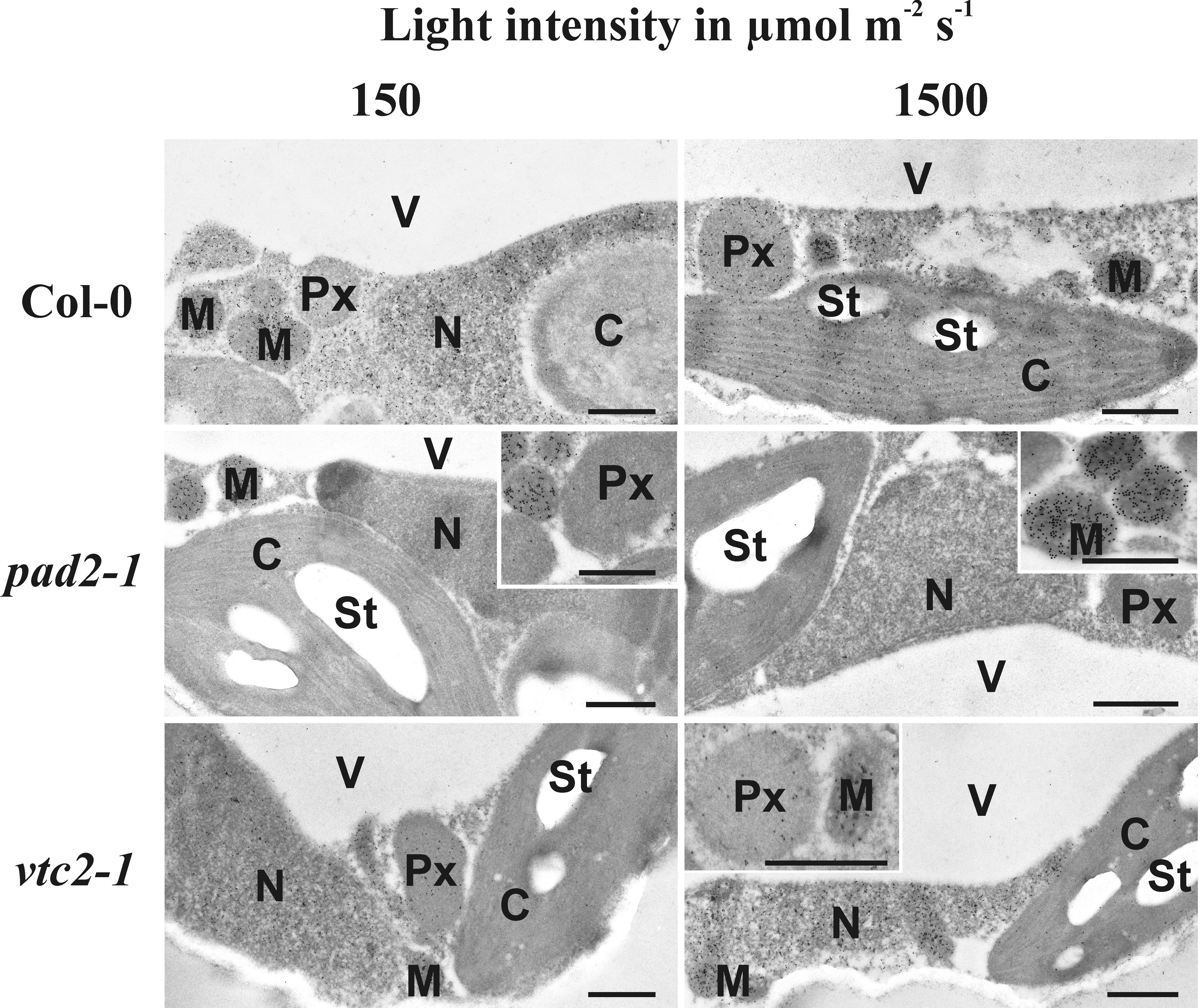

Supplement: Additional file 5 — TEM-micrographs of glutathione labeling in plants grown under different light regimes for 4 h. Representative transmission electron micrographs showing gold particles bound to glutathione on leaf sections from Arabidopsis thaliana Col-0 (first row), and the mutants pad2-1 (second row) and vtc2-1 (third row) grown under different light regimes for 4 h. Bars = 1 μm. C = chloroplasts with or without starch (St), M = mitochondria, N = nuclei, Px = peroxisomes, V = vacuoles. [file 1471-2229-13-104-S5.jpeg]

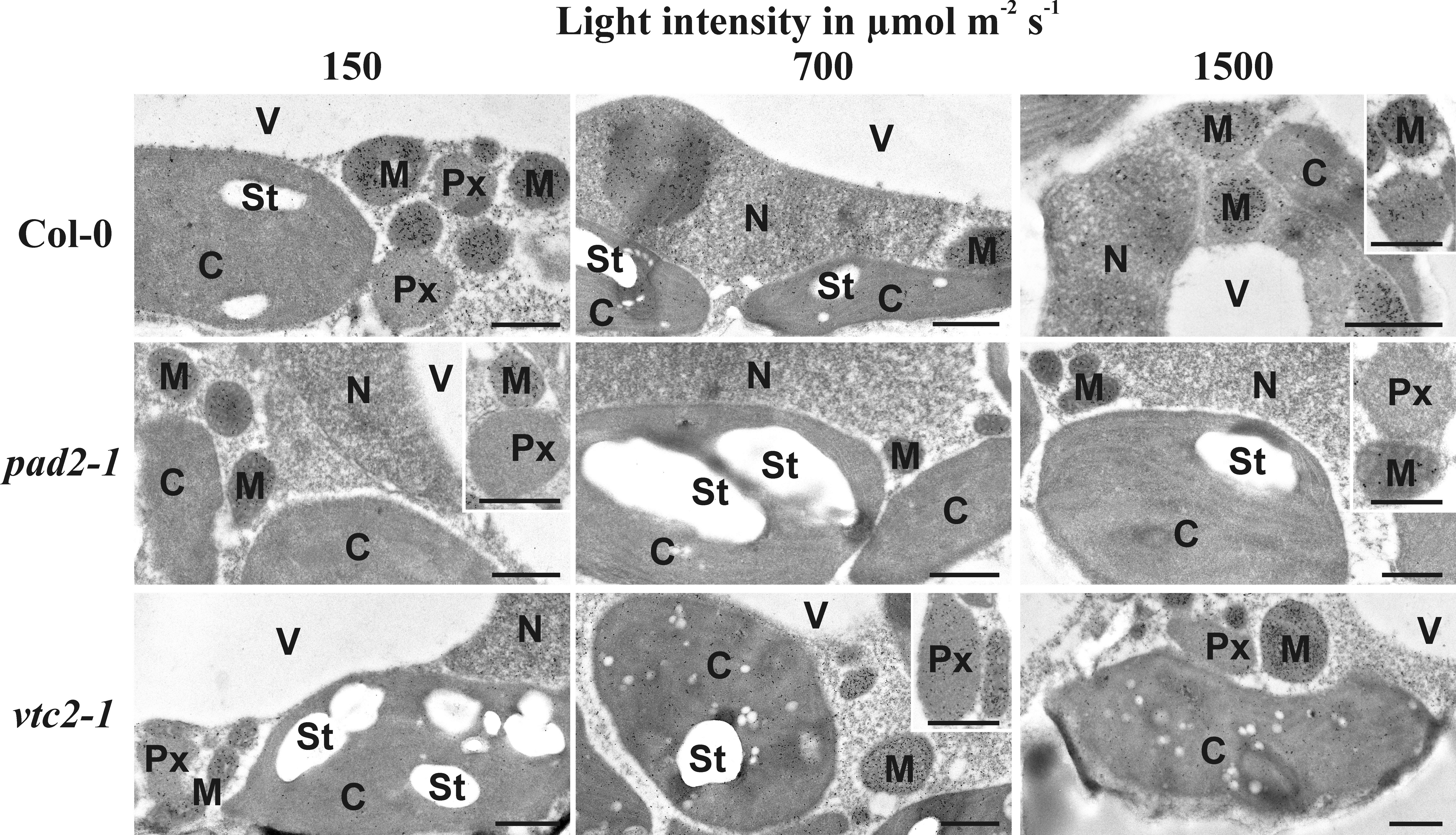

Supplement: Additional file 6 — TEM-micrographs of glutathione labeling in plants grown under different light regimes for 14 d. Representative transmission electron micrographs showing gold particles bound to glutathione on leaf sections from Arabidopsis thaliana Col-0 (first row), and the mutants pad2-1 (second row) and vtc2-1 (third row) grown under different light regimes for 14 d. Bars = 1 μm. C = chloroplasts with or without starch (St), M = mitochondria, N = nuclei, Px = peroxisomes, V = vacuoles. [file 1471-2229-13-104-S6.jpeg]

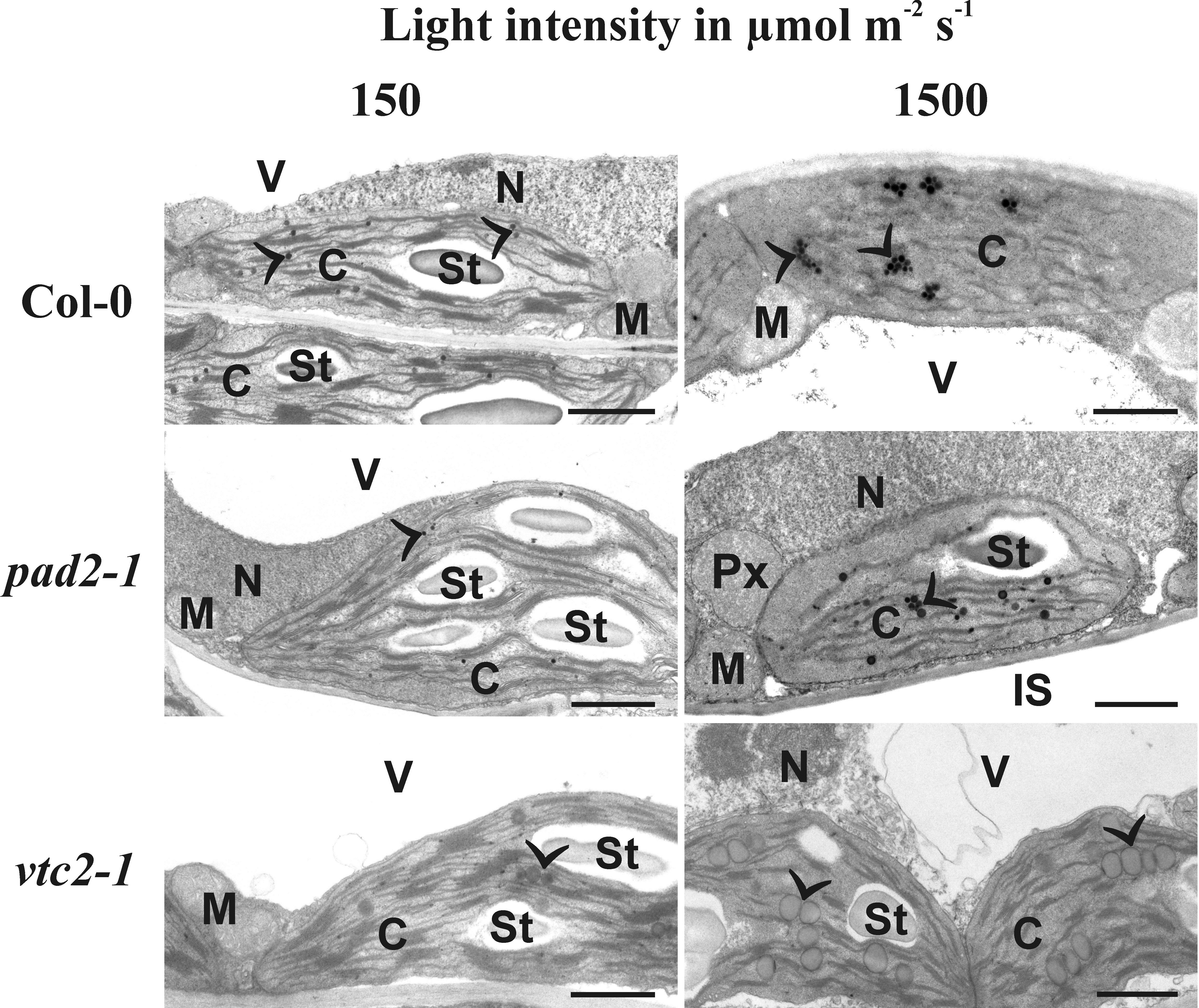

Supplement: Additional file 7 — TEM-micrographs of chloroplasts from plants grown under different light regimes for 14 d. Representative transmission electron micrographs of chloroplasts from Arabidopsis thaliana Col-0 (first row), and the mutants pad2-1 (second row) and vtc2-1 (third row) grown under different light regimes for 14 d. Bars = 1 μm. C = chloroplasts with or without starch (St) and plastoglobuli (arrowheads), IS = intercellular spaces, M = mitochondria, N = nuclei, Px = peroxisomes, V = vacuoles. [file 1471-2229-13-104-S7.jpeg]
